# New insights into nanotherapeutics for periodontitis: a triple concerto of antimicrobial activity, immunomodulation and periodontium regeneration

**DOI:** 10.1186/s12951-023-02261-y

**Published:** 2024-01-04

**Authors:** Jiaxin Li, Yuxiao Wang, Maomao Tang, Chengdong Zhang, Yachen Fei, Meng Li, Mengjie Li, Shuangying Gui, Jian Guo

**Affiliations:** 1 Department of Pharmacy, Anhui University of Chinese Medicine, Hefei, 230012 Anhui China; 2 Institute of Pharmaceutics, Anhui Academy of Chinese Medicine, Hefei, 230012 Anhui China; 3 Anhui Province Key Laboratory of Pharmaceutical Preparation Technology and Application, Hefei, 230012 Anhui China; 4 Engineering Technology Research Center of Modernized Pharmaceutics, Anhui Education Department, Hefei, 230012 Anhui China

**Keywords:** Periodontitis, Nanotherapeutics, Antimicrobial activity, Immunomodulation, Periodontium regeneration, Synergistic therapy

## Abstract

Periodontitis is a chronic inflammatory disease caused by the local microbiome and the host immune response, resulting in periodontal structure damage and even tooth loss. Scaling and root planning combined with antibiotics are the conventional means of nonsurgical treatment of periodontitis, but they are insufficient to fully heal periodontitis due to intractable bacterial attachment and drug resistance. Novel and effective therapeutic options in clinical drug therapy remain scarce. Nanotherapeutics achieve stable cell targeting, oral retention and smart release by great flexibility in changing the chemical composition or physical characteristics of nanoparticles. Meanwhile, the protectiveness and high surface area to volume ratio of nanoparticles enable high drug loading, ensuring a remarkable therapeutic efficacy. Currently, the combination of advanced nanoparticles and novel therapeutic strategies is the most active research area in periodontitis treatment. In this review, we first introduce the pathogenesis of periodontitis, and then summarize the state-of-the-art nanotherapeutic strategies based on the triple concerto of antibacterial activity, immunomodulation and periodontium regeneration, particularly focusing on the therapeutic mechanism and ingenious design of nanomedicines. Finally, the challenges and prospects of nano therapy for periodontitis are discussed from the perspective of current treatment problems and future development trends.

**Graphical Abstract:**

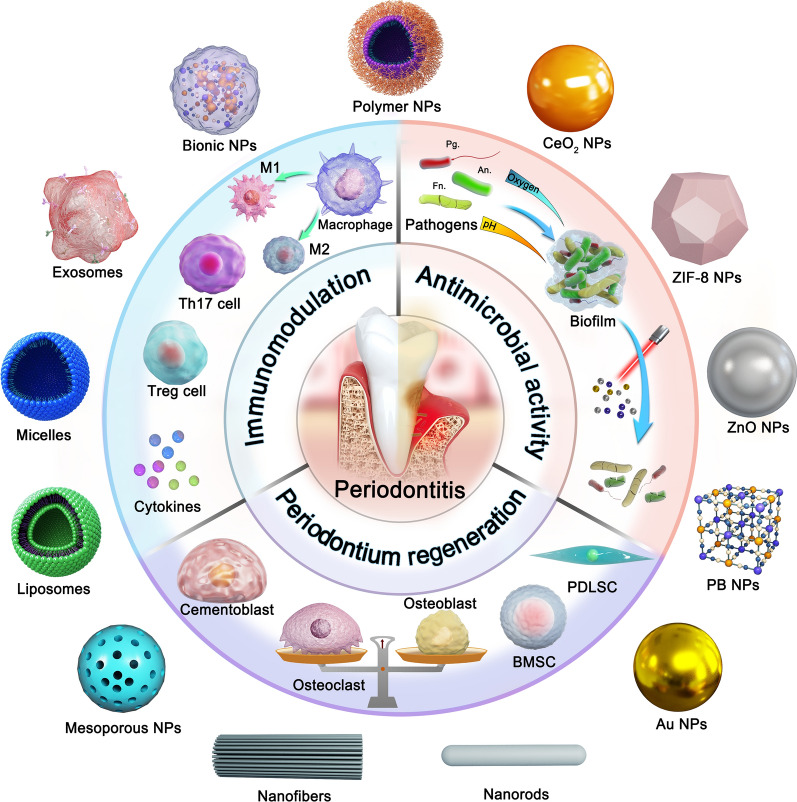

## Introduction

Periodontitis is a chronic inflammatory disease with clinical manifestations such as bleeding gums, recurrent swelling and pain, and resorption of alveolar bone. If inadequately treated, it can lead to loosening and loss of teeth, chewing function, and even effects on the digestive system [1]. In the world, periodontitis affects 11% of the global population, with prevalence rates ranging from 8 to 46% in developing countries and 3–18% in developed countries [2]. Periodontitis is more common in the aged due to chronic and cumulative injury. Two thirds of people over 65 years old in the United States have chronic periodontitis [3]. In recent decades, the incidence and prevalence of severe periodontitis in Asian countries, such as India, China and Japan, has been on the rise, and age may be a critical factor for the increasing trend [4]. In addition, there is growing clinical evidence that periodontitis places a huge burden on the public health care system due to the close links to other diseases such as diabetes, Alzheimer's disease, rheumatoid arthritis, colitis and even cancer [5–7].

Most patients with periodontitis need to be treated with scaling and root planning to remove plaque or calculus [8]. However, clinical studies have shown that scaling and root planning cannot adequately alter the composition of the subgingival microorganism biofilm, thus nonsurgical adjunctive drug therapies have been proposed to enhance treatment outcomes including the local delivery of drugs, systemic antibiotics and systemic host modulation agents. Several meta-analyses showed that systemic antibiotics and systemic host modulation agents are beneficial for periodontitis control [9, 10]. In the clinic, broad-spectrum antibiotics can be used alone or in combination with other antibiotics for Gram-negative bacteria within 1–3 weeks [11]. The long-term use of systemic antibiotics and host modulation agents may be associated with the risk of antibiotic resistance, microbial disorders and drug interactions. Local delivery of drugs into periodontal pockets is beneficial in treating patients with deep pockets or recurrent periodontitis, and administration options include nanoparticle, hydrogel, powder and fiber delivery systems.

**Fig. 1:**
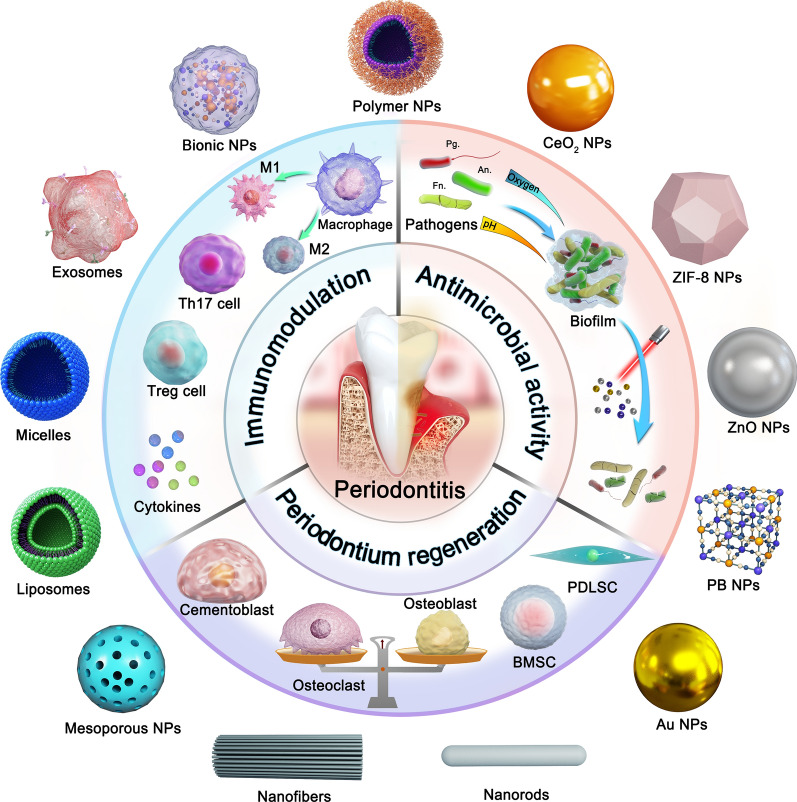
Schematic overview of nanotherapeutics via triple concerto of antimicrobial activity, immunomodulation and periodontium regeneration

Nanoparticles have significant promise for addressing the challenges of periodontal drug delivery. Nanoparticles, including liposomes, polymeric nanoparticles, polymeric micelles and nanofibers, provide great flexibility in changing chemical composition, size, surface charge and other characteristics, which can ensure stable cell targeting and oral retention. Existing data suggest that nanoparticles protect drugs from pH influence and enzymatic degradation in the periodontal lesion [12]. Importantly, the nanoparticle structure can be designed to respond to reactive oxygen species (ROS), pH, or enzyme-response mechanisms in the pathological microenvironment for controlled drug release. We summarize nano delivery systems strategies for the treatment of periodontitis in terms of three main aspects: antibacterial therapy, immunomodulatory therapy and tissue regeneration (Fig. 1). In addition, the high surface area to volume ratio of nanoparticles enables high drug or drug combination loading, resulting in synergistic therapeutic efficacy by combining multiple treatment aspects. For example, metal nanoparticles such as silver, gold, and cerium, can directly sterilize or enhance drug water solubility for transport to bacteria, simultaneously eliminating bacterial resistance through the combination of photodynamic therapy. The nanoparticles release natural active ingredients (quercetin, baicalin, caffeic acid phenethyl ester) into the periodontal pocket to reverse excessive immune response or repair the damaged periodontal tissue [13, 14]. Wu et al. used baicalein-loaded mesoporous Prussian blue nanoparticles to develop an antimicrobial and immunomodulatory synergistic nanotherapeutic strategy for the treatment of periodontitis [15]. Novel nanoparticle delivery systems, such as cell membrane biomimetic nanoparticles and exosomes, will further expand the available strategies for using nanoparticles in periodontitis treatment. In this context, we present a systematic review of the application of nanodelivery systems in the treatment of periodontitis. This review will highlight the treatment strategies and introduce the rational design of nanodelivery systems. This review will also discuss the future challenges and research directions of nano systems for periodontitis treatment.

## Pathophysiology of periodontitis

**Fig. 2:**
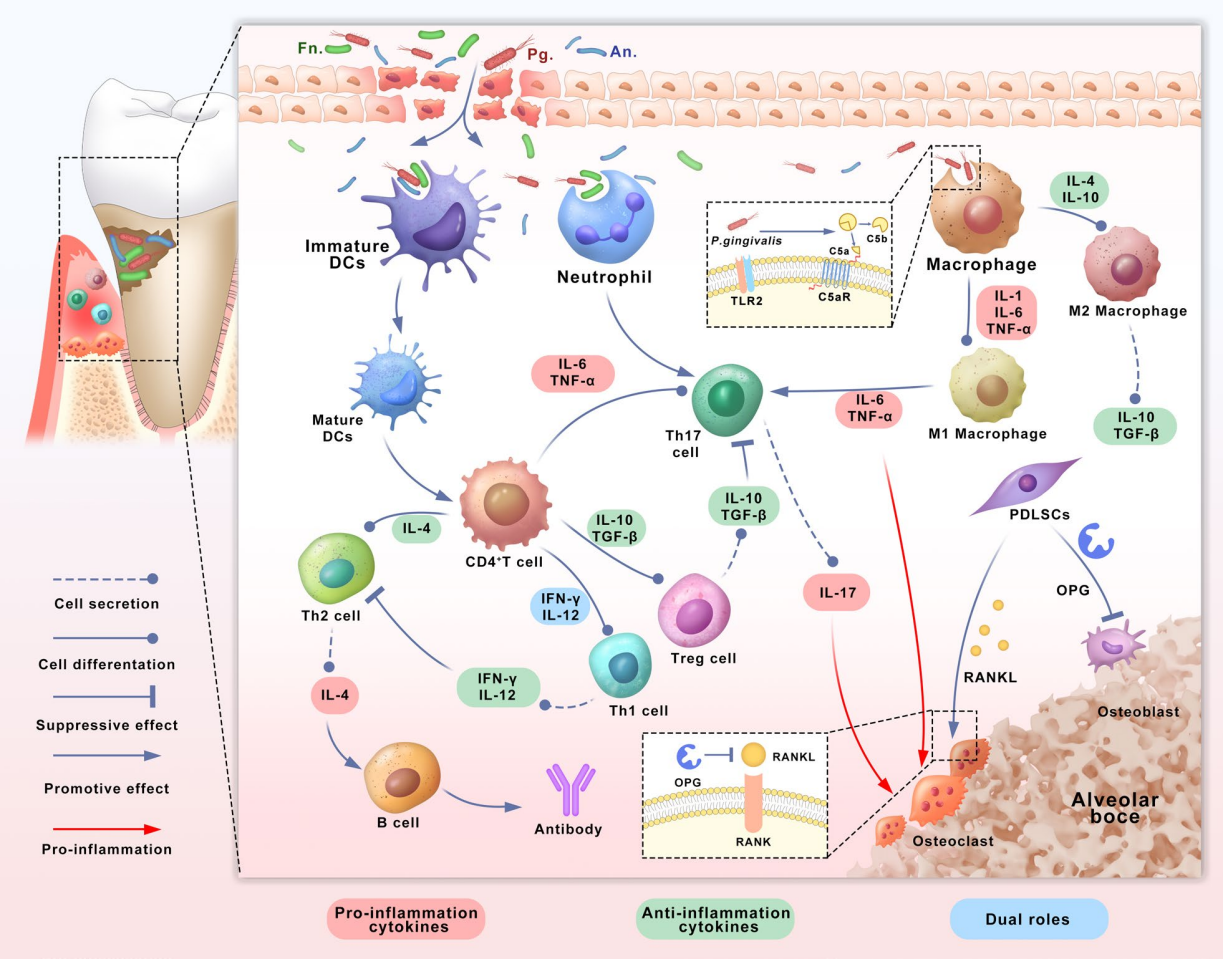
The pathogenic factors of periodontitis

An introduction to the pathological background of periodontitis is necessary for the understanding of nanotherapeutic strategies. In healthy periodontal tissue, there is a state of homeostatic balance between the microbial community and host immunity involving a minimal number of neutrophils, plasma cells, etc. [16, 17]. However, in periodontitis tissues, intractable pathogenic biofilms are present accompanied by a persistent inflammatory response and alveolar bone damage (Fig. 2). The pathogenesis of periodontitis is not completely clear, but studies have confirmed that genetic factors and plaque biofilms are underlying inducing factors. The pathogenic bacteria related to periodontitis include *Porphyromonas gingivalis* (Pg), *Prevotella intermedia*, *Actinobacillus actinomycetemcomitans*, *Fusobacterium nucleatum* (Fn), *Tannerella forsythia*, and *Treponema denticola*, *Treponema, Prevotella*, *Selenomonas*, *Peptostreptococcus*, *Anaeroglobus*, *Desulfobulbus speciesand Lachnospiraceae* [18, 19]. As many as 800 different species have been identified in dental plaque [20]. The above bacteria gather and adhere on the tooth surface to form a structured community, which is called dental plaque biofilm. Pathogenic bacteria can colonize the surface of teeth by releasing exoenzymes to produce exopolysaccharide from sucrose, which accelerates local colonization of microorganisms and establishes a meshwork of biofilm matrix [21]. The dental plaque biofilm matrix is composed of complex polymeric substances such as exopolysaccharides (glucan, fructan polysaccharides), proteins (amyloids, adhesions, DNA binding proteins), eDNA, proteoglycans, glycoproteins (Slayers, adhesins), lipids, lipopolysaccharides (LPS) and other macromolecules derived from microorganisms or hosts [20]. Meanwhile, the acidic microenvironment (pH 4.5–5.5) of the tooth surface is formed by the fermentation of bacteria within the biofilm matrix, which leads to a flourishing proliferation of pathogenic bacteria [21]. Identifying these intricate pathogenic processes may provide new opportunities for periodontitis treatment by dental biofilm control. It is worth noting the plaque biofilms are not the decisive factor for periodontal damage. Although bacterial infection is a necessary condition for the development of periodontitis, the immune response of the host is crucial for the development and tissue destruction of periodontitis [22]. The clinical consensus suggests that 80% of tissue destruction is caused by inflammatory activation via host immunity [23].

Neutrophils, as the first line of nonspecific immunity against pathogenic bacteria, attempt to engulf or kill pathogens, but they are overwhelmed by a large number of pathogens in periodontitis [24]. As antigen-presenting cells, dendritic cells (DCs) bridge the innate and adaptive immune responses in periodontitis [25]. Immature DCs have a high phagocytosis capacity and are able to rapidly capture invading microorganisms [26]. Mature DCs present antigens to the initial CD4^+^ T cell [22]. The differentiation of initial CD4^+^ T cells into T helper (Th)1, Th2, Th17 and regulatory T (Treg) cells [27]. Interferon‐γ (IFN‐γ) and interleukin (IL)-12 secreted by DCs induce Th1 cell formation in the inflammatory environment [28]. Studies have revealed that Th1 cells are involved in the progression of osteoclastogenesis and alveolar bone loss. Th2 cells are induced to form by IL-4, and IL-4 secreted by Th 2 cells further leads to B cell activation and antibody production [29]. IFN-γ secreted by Th1 cells can block the proliferation of Th2 cells, and high concentrations of IL-4 can also block the generation of Th1 cells [30]. In addition, depending on the microbial signal received by pattern recognition receptors, DCs promote Th 17 or Treg cells differentiation by releasing the cytokines of tumor necrosis factor α (TNF-α)/IL-6 or transforming growth factor β (TGF-β)/IL-10, respectively [31, 32]. Th17 cells can trigger pro-inflammatory signals, recruit and activate neutrophil granulocytes, upregulate the expression of antimicrobial factors, and promote the clearance of extracellular bacteria [33]. Moreover, activated Th17 cells secrete IL-17 to activate the Janus Kinase/signal transducer and activator of transcription signaling cascade and increase the expression of receptor activator of nuclear factor kappa-Β ligand (RANKL), eventually leading to accelerated osteoclastogenesis [34]. Activated Treg cells produce the anti-inflammatory cytokines IL-10 and TGF-β, which inhibit the Th17-related response and contribute to the maintenance of immune homoeostasis [35]. Immune disorders also promote macrophage aggregation and M1-type polarization [36]. M1-type macrophages exacerbate inflammation by secreting monocyte chemotactic proteins, IL-6, TNF-α and ROS. In addition, M1-type macrophages further act on Th17 cells, eventually leading to bone tissue injury [37, 38]. At the same time, the gingiva, cementum and periodontal ligament in the periodontium also suffer from similar destructive effects, which together lead to the aggravation of periodontitis.

## Nanotherapeutic strategies for periodontitis

Pathogenic bacteria are the initial factor in periodontitis, and the overactivated host immune response is the determinant of periodontitis exacerbation. The imbalance of pathogenic bacteria and host immunity leads to the persistence of inflammation and the destruction of periodontal tissues. Therefore, antibacterial agents, immune regulation and periodontium regeneration are the main strategies for periodontitis treatment.

### Antimicrobial nanotherapeutic strategies

Antimicrobial therapy for periodontitis has been a topic of constant discussion among researchers. Antibiotics are an important therapeutic strategy for periodontitis. However, high doses of antibiotics may create resistance problems, decreasing their therapeutic effect in the long term [39, 40]. Therefore, the development of innovative antimicrobial strategies is significant importance. In recent decades, nanoparticle delivery systems designed to overcome antibiotic resistance or metallic nanoparticles acting alone as effective antimicrobial agents have been developed for antimicrobial treatment in periodontitis therapy. In addition, nanomaterials or nanodelivery systems loaded with photosensitizers can exert bactericidal effects through photothermal therapy (PTT) and photodynamic therapy (PDT). The current nanoparticle-based antibacterial, immune regulation and periodontium regeneration treatment strategies for periodontitis are summarized (Table 1).

**Table 1:** Antimicrobial nanotherapeutic strategies for periodontitis treatment

Nanoparticles	Delivery systems	Drugs	Outcome	References
MIN-NPs	Polymeric nanoparticles	Minocycline	Clinical periodontal parameters (periodontal pocket depth, plaque index and gingival index) were significantly improved	[45]
MOX-PLGA nanoparticle	Polymeric nanoparticles	Moxifloxacin hydrochloride	Prolonged retention and drug release of a nano-loaded moxifloxacin in situ gel system in periodontal pockets	[46]
Ciprofloxacin hydrochloride loaded dual corona vesicles	Vesicles	Ciprofloxacin hydrochloride	Used double crown vesicles as drug carriers, 50% of the normal dose of antibiotics can achieve antibacterial purposes, which will reduce the possibility of antibiotic resistance	[47]
AuNCs	Au nanoclusters	/	Reduce the possibility of bacterial resistance	[50]
PCL-OTCz	Polymeric nanoparticles	Oxytetracycline hydrochloride and ZnO NPs	Excellent antibacterial activity of oxytetracycline hydrochloride in synergy with ZnO against mixed cultures of Gram-negative anaerobic bacteria	[64]
Mino-ZnO@Alb NPs	Nano-albumin	ZnO NPs and minocycline	Synergistic antibacterial action, reduced the dosage of Minocycline and avoiding the development of drug resistance	[65]
Gel MA-Au NBPs@SiO_2_	Au NBPs	Minocycline	Eliminated periodontal pathogens in periodontal pockets, photothermal treatment to maintain low bacterial retention after medication	[68]
Fe_3_O_4_-silane@Ce6/C6 MNPs	Polymeric delivery system	Ce6 and C6	Strong antibacterial activity against plaque biofilms	[73]
NaYF4-Mn@Ce6@silane	Polymeric nanoparticles	Ce6	Realized the conversion of light emission and enhance the PDT effect	[74]
TAT-Ce6/TDZ NPs	Polymeric nanoparticles	Ce6	Promoted the penetration of the bacterial cell membrane through the mediation of TAT peptide	[76]
sPDMA@ICG NPs	Polymeric delivery system	ICG	Significantly promoted the adsorption and penetration of ICG into bacterial cells, exhibiting synergistic antibacterial properties of PTT and PDT	[79]

#### Antibiotic

Antibiotics can induce bacterial death by inhibiting protein synthesis, interfering with deoxyribonucleic acid synthesis or disrupting the cell wall [40, 41]. For example, tetracyclines (minocycline, hygromycin hydrochloride) can attach to the 30S ribosomal subunit of bacteria and thus inhibit protein synthesis. Quinolones (moxifloxacin hydrochloride, ciprofloxacin hydrochloride) can interfere with deoxyribonucleic acid synthesis by inhibiting gyrase activity, causing irreversible damage to bacterial chromosomes [42, 43]. The use of nanomaterials as carriers for loading antibiotics can reduce the dose of antibiotics administered, reduce the frequency of administration and decrease the likelihood of bacterial resistance, which is an effective strategy for the treatment of periodontitis.

Yao and colleagues prepared poly (ethylene glycol)-poly (lactic acid) nanoparticle-loaded minocycline delivery systems (MIN-NPs) using an emulsion/solvent evaporation method. Nanoparticles can penetrate deeply into the periodontal pocket below the gingiva due to their small size, thus improving the antimicrobial efficacy of topical administration [44, 45]. MIN-NPs can provide sustained drug release, reduce the frequency of administration and avoid burst release of the drug. The pharmacokinetic profile showed that MIN-NPs had the longest duration of action in the gingival sulcus, compared to minocycline hydrochloride ointment and minocycline solution. The concentration of MIN-NPs in the gingival sulcus decreased slowly, maintaining the drug at an effective concentration (1.28 μg/mL) after 12 days. In vivo pharmacodynamics in beagle dogs with periodontitis showed significant improvements in periodontal pocket depth, plaque index and gingival index after 6 days of topical administration.

Moxifloxacin hydrochloride has also been used within nanosystems to improve the efficacy of periodontitis. Sarwar Beg et al. prepared poly (D, L-lactide-co-glycolide) (PLGA) nanoparticles loaded with moxifloxacin hydrochloride (MOX-PLGA) into a poloxamer 407 hydrogel to produce an in-situ gel [46]. The in vitro release profile results showed that the nanoparticles loaded into the in-situ gel resulted in a much lower drug release of 20% in the first 12 h compared to the group without gel incorporation, indicating that the gel greatly reduced the burst release of the drug. In vivo gamma scintigraphy showed that the drug in MOX-PLGA remained in the periodontium without entering the body circulation in contrast to the moxifloxacin hydrochloride solution, which facilitated the local antimicrobial effect of moxifloxacin hydrochloride in the periodontium.

**Fig. 3:**
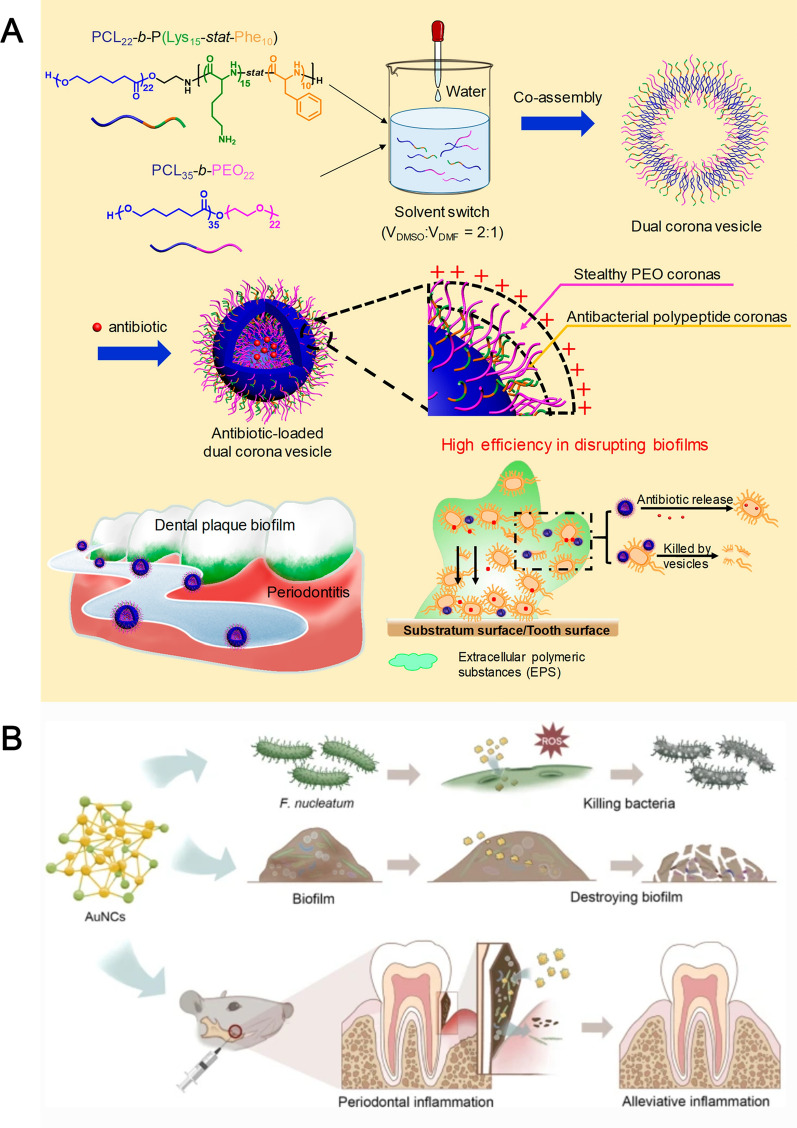
Schematic illustration of periodontitis treatment by antimicrobial and metallic nano-antibacterial agent. **A** Schematic diagram of ciprofloxacin hydrochloride-loaded dual corona vesicles. Reprinted with permission from Ref. [47] Copyright American Chemical Society. **B** Schematic diagram of AuNCs with antimicrobial nanotherapeutic strategies. Reprinted with permission from Ref. [50] Copyright Springer Nature

Xi and his colleagues proposed the encapsulate the ciprofloxacin hydrochloride in dual corona nanovesicles to reduce the antibiotic dose and antimicrobial resistance (Fig. 3A) [47]. The PCL_22_-b-P(Lys_15_-stat-Phe_10_) corona vesicles were synthesized by Z-Lys-NCA and Phe-NCA monomers. Then, PEO corona vesicles of the block copolymer using ε-caprolactone were prepared and PEO_22_-OH. PCL_22_-b-P(Lys_15_-stat-Phe_10_) and PEO were co-assembled into PEO/polypeptide dual corona vesicles by the solvent-switch method. The antibacterial polypeptide P(Lys-stat-Phe)] coronas provide vesicles with positive charges to attach to the bacterial surface. PEO coronas exhibit protein exclusion ability and penetrate extracellular polymeric substances to help antibiotics penetrate biofilms. The results showed that the minimum biofilm clearance concentrations of ciprofloxacin hydrochloride for *Escherichia coli* and *Staphylococcus aureus* biofilms were 15.36 μg/mL and 61.44 μg/mL, respectively, while the CIP-loaded dual corona vesicles decreased to 7.50 μg/mL and 30 μg/mL, respectively. In addition, scanning electron microscopy (SEM) images showed that the biofilm structure changed from tight to sparse and that the number of bacteria decreased dramatically. Nearly 70% bacteria could be killed at1 hour.

#### Metallic nano-antibacterial agent

The misuse of antibiotics in the treatment of periodontitis is a growing concern due to the problem of bacterial resistance in global public health. Therefore, there is an urgent need for new alternatives to replace traditional antibacterial therapy, and the development of metallic nanomaterials has created a new opportunity for antibacterial therapy. Among them, gold, silver, titanium dioxide, zinc oxide (ZnO) and other nano-antibacterial agents have great potential in antimicrobial therapy. In this section, we introduce only the representative antibacterial properties of metallic nano-antibacterial agents. The diverse therapeutic effects of metal nanoparticles will be further introduced in the chapter on synergistic nanotherapeutic strategies.

Compared to antibiotics, Au nanoparticles (AuNPs) exhibit unique physical properties, which include the ability to interact directly with phospholipid bilayer, bind to cytoplasmic proteins, form ROS, and thus produce significant antibacterial activity [48, 49]. Recently, researchers have tried to reduce the toxicity of Au nanoparticles by controlling their size or redesigning their shape and surface chemistry, making them better for use in medicine. Ultra-small gold nanoclusters (AuNCs) were prepared by a simple one-pot method, which composed of 25 Au atoms and 18 thiolate ligands with ultra-small structure (Fig. 3B) [50]. High-resolution-transmission electron microscopy (TEM) showed that AuNCs exhibited roughly spherical shapes with a homogeneous and well-dispersed distribution, and the particle size of AuNCs ranged from 1.5 to 4.0 nm with an average diameter of 2.49 ± 0.30 nm. The zeta potential of AuNCs was − 38.8 mV. The results showed that the growth of Fn was significantly inhibited by AuNCs at a concentration of 0.2 mM, and cell wall integrity was damaged. These results indicated that AuNCs induced lysis of the Fn membrane potential is crucial for bacterial energy metabolism and is an early sign of membrane damage. The red/green fluorescence ratios were reduced by approximately 50% after AuNCs incubation, and the membrane potential reduction indicated that AuNCs treatment caused serious damage to cell membranes, possibly inhabiting bacterial growth via a membrane depolarization mechanism. In addition, the level of ROS in Fn was enhanced after AuNCs treatment. It would be difficult for bacteria to develop resistance to AuNCs based on their antimicrobial mechanisms.

ZnO nanoparticles have excellent safety and biocompatibility with multiple antibacterial mechanisms, in the tissue engineering, food packaging and dental materials fields with a wide range of roles [51–53]. The antibacterial mechanisms of ZnO nanoparticles include (1) electrostatic interaction with the cell wall [54–56] (2) adsorption of Zn^2+^ on bacterial surfaces, altering membrane permeability [57–59] (3) production of ROS to exert antibacterial effects [60, 61], and ZnO nanoparticles thus have enormous potential in periodontitis treatment. Eliseu A. Münchow and his colleagues loaded ZnO nanoparticles onto poly(has-lactide)/gelatine blend fibrous membranes by electrostatic spinning [62]. The results of agar diffusion experiments showed that the loaded ZnO nanoparticles inhabited Pg and Fn with inhibition zones ranging from 6 to 15 mm in diameter, showing beneficial antibacterial effects. Furthermore, it was reported that the electrospun polymer film loaded with ZnO not only has antibacterial properties, but its production of reactive oxygen species can promote cell proliferation and wound healing mediated by growth factors, which offers the possibility of treating periodontitis [63].

The combination of antibiotics and ZnO nanomaterials has become an actively explored strategy for periodontitis treatment. For instance, the polycaprolactone nanofibers were selected as drug carriers loaded with oxytetracycline hydrochloride and ZnO nanoparticles (PCL-OTCz) [64]. Four Gram-negative anaerobic bacteria (Pg, *Aggregatibacter actinomycetemcomitans*, *Prevotella intermedia*, *Treponema denticola*) were selected to form mixed bacterial cultures. PCL-OTCz showed powerful antibacterial activity compared with oxytetracycline hydrochloride or ZnO nanoparticles, individually. In another study, ZnO nanoparticles and minocycline were coloaded on a nano-albumin carrier (Mino-ZnO@Alb NPs) and incorporated into a pH-responsive Carbopol 940 hydrogel [65]. Mino-ZnO@Alb NPs showed pH-responsive characteristics with 80% release of minocycline at pH 6.5, while only approximately 40% of minocycline was released at pH 8.5, probably due to higher minocycline partitioning under acidic conditions. The results of the inhibition circle method showed that Mino-ZnO@Alb NPs could significantly inhibit the bacterial proliferation of Pg, *Streptococcus oralis*, *Streptococcus sanguis* and Periodontopathic bacteria. These results indicate that the combination of zinc oxide nanoparticles and antibiotics is a potential antibacterial strategy for the treatment of periodontitis.

#### Photothermal/photodynamic therapy

PTT and PDT have been proposed for the antimicrobial treatment of periodontitis. In comparison to conventional antibiotic treatment, PTT and PDT are less likely to cause resistance in periodontitis-causing bacteria. Second, the wavelength of the light source used for photothermal antimicrobial treatment has deeper tissue penetration than that of other light-induced treatments, allowing it to penetrate deeper into the periodontium [66, 67]. Finally, by controlling the specific area and intensity of light exposure, the side effects of photothermal antimicrobial treatment on normal tissues can be reduced.

PTT antimicrobial therapy uses photothermal material to produce a lethal effect on bacteria by heating up under irradiation. Lin et al. explored a PTT therapeutic strategy by first synthesizing a near-infrared responsive photothermal material of Au nano bipyramids (Au NBPs) [68]. Then minocycline-loaded mesoporous silica was modified on the Au NBPs surface, and Au NBPs@SiO_2_ were finally mixed with gelatine methacrylate to prepare a hybrid hydrogel (Gel MA-Au NBPs@SiO_2_). The temperature of Au NBPs@SiO_2_ increased under near infrared (NIR) irradiation. Furthermore, the diffusion and release of minocycline accelerated with increasing power of irradiation. Compared to non-NIR irradiation, the antibacterial rate of Gel MA-Au NBPs@SiO_2_ against Pg increased to 66.7% via the increase in NIR irradiation power. The live/dead staining results showed that the antibacterial effect of Pg was up to 90% on the third day but only 10% on the seventh day, while the antibacterial effect could be increased to 50% on the eighth day after NIR treatment. This suggests that light and heat can cooperate with antibiotics for antibacterial treatment without developing resistance.

PDT antimicrobial therapy refers to the use of a photosensitizer that produces ROS in response to light stimulation, thus leading to rapid oxidation of lipids in bacteria, and the destruction of fragile lipid membranes in periodontitis treatment [69, 70]. Chlorin e6 (Ce6) is widely used in PDT therapy because of its strong tissue penetration, great biocompatibility and high yield of singlet oxygen [71, 72]. Sun et al. prepared a core–shell structure nanodrug delivery system (Fe_3_O_4_-silane@Ce6/C6) including Fe_3_O_4_ nanoparticles, Ce6 and coumarin 6, for PDT antimicrobial therapy [73]. Transmission electron microscopy (TEM) images of Fe_3_O_4_ nanoparticles show a diameter of approximately 8 nm and Fe_3_O_4_-silane@Ce6/C6 a diameter of about 100 nm. Fe_3_O_4_-silane@Ce6/C6 produced O_2_^−^ under light irradiation and had a killing effect on *Streptococcus sanguis*, Pg and Fn Under light irradiation, Fe_3_O_4_-silane@Ce6/C6 reduced Fn and Pg biofilm colony formation units by approximately 4 log and 5 log, respectively. Notably, Fe_3_O_4_-silane@Ce6/C6 can be magnetically induced to concentrate at plaque biofilm sites to further enhance the antibacterial effect. These results indicate that PDT combined with magnetic targeted nanoparticles has potential for antimicrobial therapy for periodontitis.

**Fig. 4:**
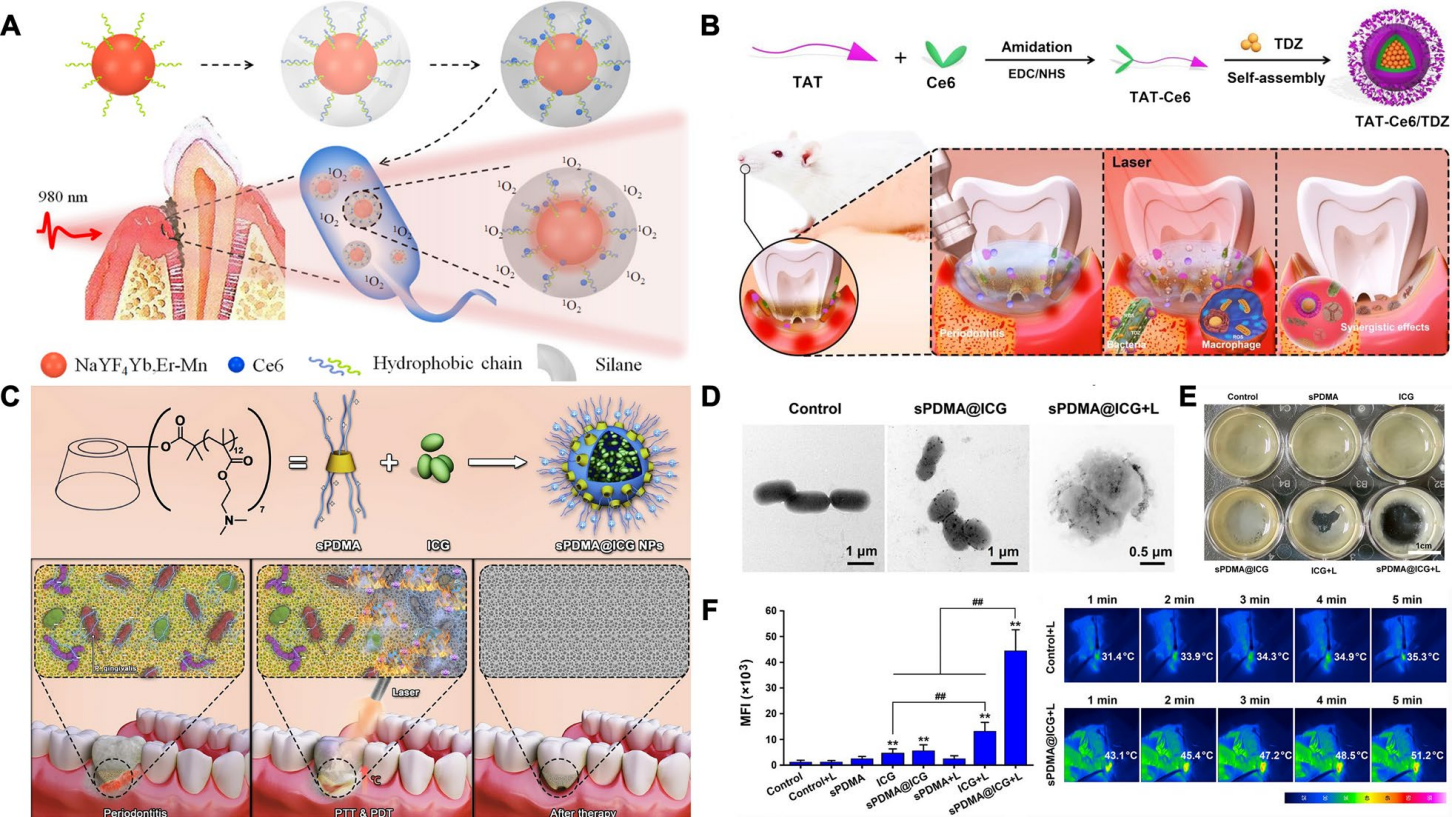
Schematic illustration of periodontitis treatment by photothermal/photodynamic therapy. **A** The schematic diagram of NaYF4-Mn@Ce6@silane. Reprinted with permission from Ref. [74] Copyright Multidisciplinary Digital Publishing Institute. **B** Preparation and against periodontitis of TAT-Ce6/TDZ NPs. Reprinted with permission from Ref. [76] Copyright American Chemical Society. **C** Preparation of sPDMA@ICG NPs and treatment of synergistic PTT and PDT on periodontitis. **D** TEM images of bacteria after processions of PBS (the control), sPDMA@ICG NPs with and without laser irradiation. **E** Photos of plaque biofilms after various processions. **F** Comparison for the mean fluorescence intensities of produced DCF in periodontium. Infrared thermal images of periodontium during laser irradiation after administration of PBS (the control) and sPDMA@ICG NPs. **indicates p < 0.01 compared to the control (+); ^##^indicates p < 0.01 for comparison between two groups. Reprinted with permission from Ref. [79] Copyright Springer Nature

Although PDT has made important progress in the treatment of periodontal disease, there are some serious problems that still need to be solved before clinical application. The most important limitation of conventional PDT is the weak tissue penetration of ultraviolet or visible light. Therefore, it is highly desirable to design and prepare a PDT system that relies on with the infrared irradiation light, which can perform deep-tissue penetration. Based on this, a strategy was proposed to combine the photosensitizer Ce6 with upconversion nanoparticles (UCNPs) NaYF4:Yb, Er [74]. The combination of Ce6 UCNPs was realized via an amphiphilic silane modification technique (NaYF4@Ce6@silane NPs), which involved the hydrophobic-hydrophobic interaction between the hydrophobic side chain of the silane and hydrophobic groups on the surface of UCNPs [75]. In addition, because the PDT function of the Ce6 molecule should be triggered by excitation by red light, Mn doping is involved in this work, which greatly improves the probability of the red emission transition (NaYF4-Mn@Ce6@silane) (Fig. 4A). The TEM image showed that the size distribution of NaYF4@Ce6@silane NPs was approximately 30 nm, and a thin layer of silane (approximately 2–3 nm) was observed on the surface of the nanoparticles after silane coating. The result showed that 30% Mn was selected to dope into UCNPs to realize the enhancement of red-light emission, and the enhanced upconversion red emission can further improve the PDT effect. The colony forming units of Pg, Fn and *P. intermedia* after NaYF4-Mn@Ce6@silane NPs with 980 nm irradiation decreased by more than 2 log, and the biofilm matrix was easily disrupted with deeper penetration of infrared light. This highly efficacy against periodontitis-related biofilms should be attributed to the high hydrophilic surface after silane modification, as well as to the upconversion luminescence triggered PDT. This upconversion PDT design can overcome the problems of conventional PDT and provide effective nano strategies for the treatment of periodontitis.

However, PDT does not always achieve the desired therapeutic outcomes since photosensitizers Ce6 have strong hydrophobic properties and are not taken up efficiently by periodontal pathogenic bacteria. Li et al. designed a nanosystem to improve Ce6 solubility and enhance its bacterial adsorption by promoting its interaction with negatively charged cell walls and penetration through cell membranes [76]. They first hydrophilically-modified Ce6 via conjugation with TAT peptide, a cationic cell-penetrating peptide (TAT-Ce6). Then, TAT-Ce6 was loaded with the antibiotic agent tinidazole to prepare self-assembled nanoparticles (TAT-Ce6/TDZ NPs) to achieve synergistic anti-periodontitis effects by combining PDT and antibiotic therapy (Fig. 4B). TEM image showed that TAT-Ce6/TDZ NPs had a regular spherical shape and exhibited a more compact inner structure. The particle size of TAT-Ce6/TDZ NPs was ~ 146.2 nm, and the zeta potential was approximately + 40.1 mV, which confirmed the surface distribution of the positively charged TAT peptide. The UV absorption of ABDA attenuates when ABDA is decomposed by the ROS produced in the sample solution, and the degree of attenuation is positively correlated with the ROS generation level. The results showed that the attenuation rate of ABDA absorption in the solution of TAT-Ce6/TDZ NPs was much faster than that of free Ce6 during 20 min of laser irradiation, indicating that TAT-Ce6/TDZ NPs had a much higher PDT efficiency. The zeta potentials of Pg were increased from − 11.2 to + 5.86 mV after 1 h of incubation with TAT-Ce6. The above results indicated that TAT-Ce6 significantly promoted the penetration of the bacterial cell membrane through the TAT peptide. TAT-Ce6/TDZ NPs and TAT-Ce6 exhibited much stronger bacterial killing activity, owing to their more efficient absorption by the plaque biofilms via the mediation of TAT peptide. More importantly, TAT-Ce6/TDZ NPs exhibited much stronger bacterial killing activity than TAT-Ce6 NPs with laser irradiation, further confirming their synergistic antibacterial efficacy through combining PDT and antibiotic therapy.

Indocyanine green (ICG), a photosensitizer with PDT properties, has been approved for clinical use by the US Food and Drug Administration [77]. Nagahara et al. first explored PDT of photosensitizer indocyanine green, which has high absorption at a wavelengths of 800–805 nm [78]. They designed ICG-loaded PLGA nanospheres coated with chitosan (ICGNano/c) and explored the PDT of ICGNano/c in Pg. The study showed that ICG-Nano/c with low-level diode laser (0.5 W, 805 nm) irradiation showed a PDT-like effect, which might be useful for potential photodynamic periodontal therapy. Recently, combined treatment with PTT and PDT has further improved the efficiency of periodontitis treatment. However, due to its negative charge and water solubility, ICG has difficulty passing through bacterial cell membranes. To address this problem, Shi and his colleagues incorporated it into positively charged polycationic brush nanoparticles (sPDMA@ICG NPs) (Fig. 4C) [79]. CD-Br was synthesized by esterifying β-cyclodextrin with 2-bromoisobutyryl bromide via an esterification reaction. Next, star-shaped polycationic brush poly (2-(dimethyl amino) ethyl methacrylate) was synthesized by an atom transfer radical polymerization reaction using CD-Br as an initiator. Finally, sPDMA@ICG NPs loaded with ICG were prepared by the nanometer precipitation method. The average particle size of sPDMA@ICG NPs was 206 nm and the zeta potential was approximately + 18.4 mV. On the one hand, the temperature of the sPDMA@ICG NPs solution was increased from 22 to 55 °C after irradiation with an 808 nm laser (2 W/cm^2^), which reflects an excellent PTT performance. On the other hand, sPDMA@ICG NPs also exert PDT properties. The results suggested that sPDMA@ICG NPs can produce ROS after laser irradiation, as detected by SOSG. Confocal microscopy images show that sPDMA@ICG NPs are effectively accumulate in bacterial cells after the administration of sPDMA@ICG NPs. TEM images showed that sPDMA@ICG NPs were clearly visible on the surface of Pg, and that the bacterial film ruptured and bacterial cells disintegrated after laser irradiation (Fig. 4D). In addition, sPDMA@ICG NPs with laser irradiation reduced the growing area of plaque biofilms derived derive from a rat model of periodontitis (Fig. 4E). After sPDMA@ICG NPs administration and laser irradiation, temperature and ROS levels were increased in rats with periodontitis, indicating that sPDMA@ICG NPs exert synergistic PTT and PDT effects in vivo (Fig. 4F).

### Immunomodulatory nanotherapeutic strategies

In periodontitis, the presence of plaque microorganisms and their products can activate the host immune response [80]. Local host immune overreaction then, leads to increased inflammation and disruption of homeostasis, exacerbating periodontium lesions. Most tissue damage within periodontitis is caused by the host immune response rather than directly by the infecting microorganism [81, 82]. Therefore, in terms of therapeutic strategies, suitable immunomodulatory targets can be screened to modulate the host immune system to mitigate the inflammatory response. Recently, many nanosystems have been designed to modulate the function of immune cells and inflammation-associated cytokines to alleviate periodontal inflammation, and these nanosystems have achieved excellent therapeutic results both in vitro and in vivo (Table 2).

**Table 2:** Immunomodulatory nanotherapeutic strategies for periodontitis treatment

Nanoparticles	Delivery systems	Drugs	Outcome	References
Lipo-RSV	Liposome	Resveratrol	Reprogramed the macrophages from M1- to M2-like phenotype, adjustment of the immune microenvironment	[89]
DPSC-Exo/CS	Exosomes	/	Suppressed periodontal inflammation by promoting the conversion of macrophages from a pro-inflammatory to an anti-inflammatory phenotype in the periodontium of mice with periodontitis	[90]
CeO_2_@QU	Nanoparticles	Quercetin	Scavenged ROS and regulated the conversion of M1 phenotype macrophages to the M2 phenotype to regulate the immune microenvironment	[14]
3D-exos	Exosomes	/	Alleviated Th17 cell/Treg imbalance and reduce inflammation	[99]
PDLSC-exos	Exosomes	/	PDLSC-exos transferred miR-155-5p into CD4 + T cells to affecting the Th17/Treg homeostasis	[100]
Nano-BA/BE	Polymeric nanoparticles	Baicalein/Baicalin	Alleviated the expression of Il-1β-induced pro-inflammatory cytokines and regulating the immune microenvironment	[104]
PDA NPs	Polydopamine nanoparticles	/	Effectively scavenges ROS in the body, relieves oxidative stress and reduces local periodontal inflammation	[105]

#### Remodeling macrophage polarization

In the immune system, immune cells such as macrophages play an important role as the host's first line of defenses against microorganisms. When induced by different factors, macrophages polarize and develop different phenotypes, such as M1 and M2, both of which are involved in regulating the immune response [83]. M1-type macrophages produce the cytokines IL-6 and TNF-α, which promote the inflammatory response [84, 85]. M2 macrophages can be further classified into alternatively activated macrophages (M2a), type 2 macrophages (M2b), deactivated macrophages (M2c), and M2-like macrophages (M2d) by different stimuli and transcription levels [86, 87]. M2a macrophages are induced by IL-4 and IL-13 and secrete profibrotic factors such as TGF-β and insulin-like growth factor, and fibronectin contributes to tissue repair. M2b cells express and secrete substantial amounts of the anti-inflammatory cytokine IL-10 and low levels of IL-12, which is the functional conversion of M1 cells. M2c macrophages are induced by IL-10 and strongly exhibit anti-inflammatory activities by releasing large amounts of IL-10 [88]. M2d macrophages are also known as tumor-associated macrophages. In a word, M2 type macrophages exert anti-inflammatory and angiogenic effects and promote tissue repair and wound healing. Modulation of macrophage phenotypes through nanosystems provides a promising therapeutic strategy for periodontitis.

**Fig. 5:**
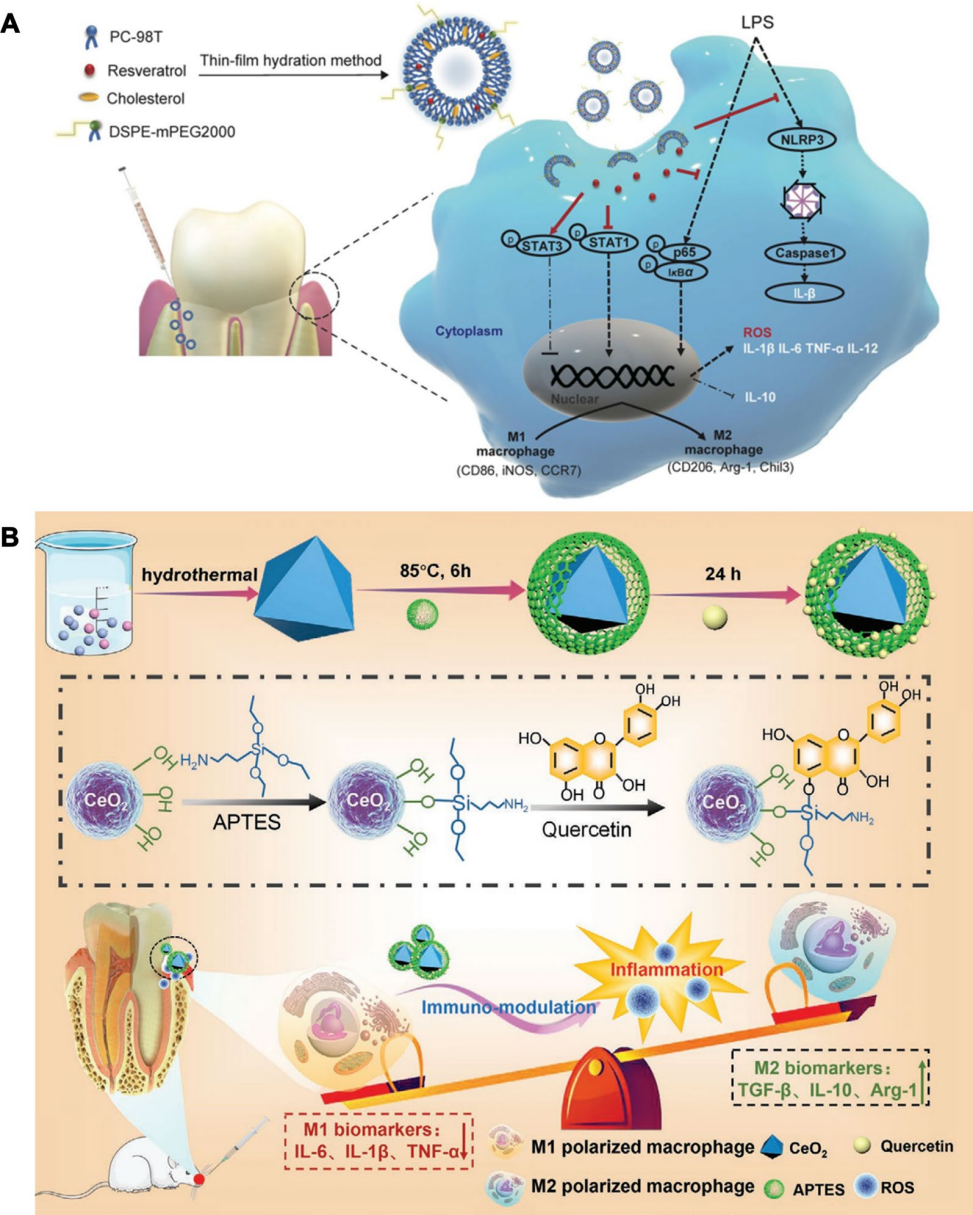
Schematic illustration of periodontitis treatment by remodeling macrophage polarization. **A** Schematic representation of Lipo-RSV regulating macrophage phenotype through activation of p-STAT3 and downregulation of p-STAT1. Reprinted with permission from Ref. [89] Copyright Springer Nature. **B** Schematic illustration of preparation and therapeutic mechanism of CeO_2_@QU nanoparticles. Reprinted with permission from Ref. [14] Copyright John Wiley and Sons

Shi et al. developed a liposome loaded with resveratrol (Lipo-RSV) to polarize macrophages from the M1 to M2 phenotype (Fig. 5A) [89]. Lipo-RSV upregulated the mRNA expression levels of M2-related markers (CD206, Arg-1 and Chil3), and downregulated the mRNA expression levels of M1 macrophage markers (CD86, iNOS and CCR7) in activated macrophages. Lipo-RSV treatment increased the percentage of M2-like subpopulations (F4/80^+^CD206^+^) by 14%, while the percentage of M1-like subpopulations (F4/80^+^CD86^+^) decreased by 8%. The mechanistic results showed that Lipo-RSV inhibited the phosphorylation of STAT1 and promoted the phosphorylation of STAT3 in bone marrow-derived macrophages. In addition, the secretion of proinflammatory cytokines (IL-1β, IL-6, TNF-α, and IL-12) and the level of ROS were attenuated, while the anti-inflammatory cytokine IL-10 was upregulated after Lipo-RSV treatment. This suggests that Lipo-RSV has the potential to treat periodontitis by regulating the polarization of M1 macrophages into M2-type macrophages.

Exosomes are nanosized (30–120 nm) vesicles secreted by various cell types that, in addition to acting as carriers to deliver drugs, may modulate macrophage phenotypes. Shen et al. isolated dental pulp stem cells from shed teeth from healthy donors, cultured them in medium and then collected their exosome pellets by centrifugation for the treatment of mice with periodontitis [90]. To enhance the stability of the exosomes, chitosan hydrogels loaded with dental pulp stem cell exosomes (DPSCs-Exo/CS) were prepared in mixed culture with chitosan hydrogels at 37 °C in a 1:1 volume ratio. Approximately 7351 genes were regulated in the periodontium of periodontitis mice after DPSC-Exo treatment. CD206^+^ cells are considered anti-inflammatory macrophages that promote the healing of periodontitis. The images showed more CD206^+^ cells in the periodontium of DPSC-Exo/CS-treated mice. After DPSCs-Exo/CS administration, the expression of anti-inflammatory markers (CD206^+^, Arg^+^) was significantly increased, and the expression of pro-inflammatory markers (CD86^+^, iNOS^+^) was downregulated. The Gene Ontology term enrichment analysis results further indicated that DPSC-Exo/CS downregulated both the inflammatory response and immune response in mice with periodontitis. To explore the underlying mechanism by which DPSC-Exo facilitates the conversion of the macrophage inflammatory phenotype, miRNA sequencing (RNA-seq) data showed that miR-1246 accounted for 43.9% of the total miRNA reads. These results suggested that DPSC-Exo/CS can promote the conversion of macrophages from a pro-inflammatory phenotype to an anti-inflammatory phenotype, and the mechanism of phenotype conversion might be linked with miR-1246 in DPSC-Exo.

ROS production is a major biological process in stimulated macrophages involved in the killing of phagocytized microorganisms [91]. However, excessive ROS may drive macrophages to the M1 phenotype to exacerbate the development of inflammation [92]. Cerium oxide (CeO_2_) is a nanoenzyme that can scavenge O_2_•^−^ and H_2_O_2_ efficiently by shifting the Ce^3+^ (reduced)/Ce^4+^ (oxidized) forms, and presenting SOD- and CAT-mimetic activities [93]. Notably, the intrinsic reductive structures, such as a catechol group, a 2,3-double bond, and hydroxyl substitution in the heterocyclic ring, allow quercetin to scavenge ROS. Therefore, Wang et al. constructed a nanocomplex (CeO_2_@QU) that integrates ROS-scavenging nanoenzymes and antioxidant natural flavonoids [14]. In their study, CeO_2_ was first prepared by a hydrothermal method; 3-aminopropyltriethoxysilane was used to amino-functionalize CeO_2_, and the addition of quercetin with stirring yielded CeO_2_@QU (Fig. 5B). CeO_2_@QU showed an octahedral morphology with an average particle size of 120 nm. The results showed fewer CD86-positive cells (40.5%) and significantly increased expression of CD206-positive cells (83.9%) after CeO_2_@QU treatment. The images showed that the expression of the M2 biomarker Arg^+^ was upregulated. Rats underwent ligature placement and bacterial injection for 3 d to establish an animal model of periodontal inflammation. The results showed low fluorescence of ROS in vivo after CeO_2_@QU treatment, and the expression of pro-inflammatory M1 biomarker IL-1β also was downregulated after CeO_2_@QU treated. These results confirmed that this system has the potential to regulate the immune microenvironment by scavenging ROS and regulating the conversion of M1 phenotype macrophages to the M2 phenotype.

#### Restoring Th17/Treg cells balance

After receiving antigenic stimulation, primary CD4^+^ T cells can differentiate into different subtypes of T cells under different conditions. Among these, the balance between Th17 and Treg cells is crucial in the periodontal immune response [94, 95]. Overreaction of Th17 cells to pathogens leads to increased expression of the pro-inflammatory cytokines IL-17 and IL-22 [96]. IL-17 has been shown to boost RANKL expression while inhibiting OPG expression in periodontal ligament cells, which might explain why Th17 cells promote alveolar bone loss [95]. However, Tregs can suppress the host immune response in equilibrium with Th17 cells in the periodontium [97, 98]. To date, no drug/nanosystem has been reported to directly regulate the Th17/Treg cells balance, but exosomes derived from periodontal ligament stem cells have been found to have the potential to regulate Th17/Treg cells.

Zhang et al. found that exosomes derived from mesenchymal stem cells (3D-exos) could regulate Th17/Tregs cell balance [99]. Notably, replacing the traditional 2D culture system with a 3D system could increase exosome production. The average particle size of the 3D-exos was 50–200 nm. A significant Th17 reduction and Treg elevation were observed in the periodontium after treatment with 3D-exos in periodontitis mice. Gene Ontology analysis showed that differentially expressed genes in 3D-exo-treated mice with periodontitis were enriched for T-cell chemotaxis. These results suggested that 3D-exos can further regulate Th17/Tregs cells in a periodontitis mice model. Furthermore, RNA-seq and TargetScan results indicated that miR-1246 is the most differentially expressed miRNA in 3D-exo, which targets Nfat5. Nfat5 is a key factor that mediates Th17 cell polarization in a sequence-dependent manner. Therefore, 3D-exo suppresses Th17 cell differentiation by miR-1246 through downregulation of Nfat5 gene expression.

Zheng et al. investigated the effect of exosomes from periodontal membrane stem cells (PDLSC-exos) on Th17/Treg balance [100]. The expression of the Th17-related transcription factor RAR-related orphan receptor C was upregulated and the Treg-related transcription factor forkhead Box P3 was down-regulated in periodontitis patients. This means that the Th17/Treg ratio is unbalanced in patients with periodontitis. The results showed lower Th17-related CD4^+^/IL‐17^+^ expression and increased Treg-related CD4^+^CD25^+^FOXP3^+^ expression after treatment of PDLSC-exos in CD4^+^ T cells, confirming the regulatory effect of PDLSCs-exos on the Th17/Treg balance. In addition, the mechanistic results showed that PDLSC-exos transfer miR-155-5p into CD4^+^ T cells, which in turn regulates the expression of histone deacetylase protein in CD4^+^ T cells, thus affecting the Th17/Treg homeostasis. Therefore, miR-155-5p may be a promising target for the treatment of immune imbalance in periodontitis. In summary, exosomes are a potential nano drug delivery system to regulate the balance of Th17 and Treg cells, and their potential in the treatment of periodontitis needs to be further explored.

#### Regulating pro-/anti-inflammatory cytokine secretion

Inflammatory cytokines secreted from immune or tissue cells are key regulators of the immune response process, and pro-/anti-inflammatory cytokine imbalance is an important factor in the aggravation of periodontitis [37]. Cytokines, such as IL-1β, IL-6 and TNF-α, activate inflammation-related transcription factors or activate related signaling pathways, thereby accelerating the process of periodontitis. The anti-inflammatory cytokines of IL-10, TGF-β and IL-11, downregulate the expression levels of pro-inflammatory factors, protect the periodontium and inhibit the development of periodontitis [101–103]. It is extremely important to modulate pro-/anti-inflammatory cytokine secretion for restore immune balance in periodontitis treatment.

Li et al. prepared baicalin and baicalein-loaded mesoporous silica nanoparticles (Nano-BA and Nano-BE) to regulate inflammatory cytokine secretion [104]. The mesoporous silica nanoparticles were modified by 3-aminopropyl-triethoxy silane. An inflammation cell model was established by primary human gingival epithelial cells pretreated with IL-1β stimulation. Nano-BA and Nano-BE downregulated cytokines involved in the immune inflammatory response. Among them, epithelial cell-derived neutrophil-activating peptide 78, monocyte chemoattractant protein-1, and IL-8 function as chemokines leading to inflammation or tissue damage, while granulocyte colony-stimulating factor and granulocyte–macrophage colony-stimulating factor stimulate the differentiation and proliferation of hematopoietic stem cell immune cells.

In another study, Polydopamine nanoparticles (PDA NPs) were synthesized via self-polymerization with dopamine hydrochloride and a solution containing NH_4_OH and ethanol [105]. PDA NPs effectively reduce the levels of TNF-α and IL-1β inflammatory mediators. After PDA NPs treatment in mice, the cytokine levels of TNF-α, IFN -γ, and IL-1β in serum recovered to normal values. Moreover, all the levels of alanine aminotransferase, aspartate aminotransferase and alkaline phosphatase (ALP) were also in the reference normal ranges. Notably, PDA NPs efficiently reduced the level of ROS in LPS-induced local high fluorescence signals. The above results suggest that reducing ROS levels may help to regulate inflammatory cytokine levels during periodontitis treatment.

### Periodontium regeneration nanotherapeutics strategies

The periodontium consists of the gingiva, periodontal ligament, alveolar bone and cementum, which provide physical and mechanical support for the teeth [106, 107]. Severe periodontitis leads to the loss of periodontal attachment, which is one of the major causes of tooth loss in adults. Therefore, the main goal of periodontitis treatment is to reduce the destruction of the periodontium, finally achieving periodontium regeneration. In recent years, researchers have proposed a series of advanced nanotherapeutic strategies to achieve periodontium regeneration by regulating cell differentiation and disturbing osteoclastogenesis (Table 3).

**Table 3:** Periodontium regeneration nanotherapeutic strategies for periodontitis treatment

Nanoparticles	Delivery systems	Drugs	Outcome	References
L-Cys-AuNPs	Au nanoparticles	/	Promoted proliferation of human PDLCs, enhances their ALP activity and upregulated mRNA levels of osteogenic genes	[112]
Nano-CaF_2_	Polymeric nanoparticles	/	Increased levels of human PDLSCs and osteogenic genes in dental bone promote osseous differentiation and contribute to periodontium regeneration	[113]
h-PDLSCs-exosomes	Exosomes	/	Upregulated of Runx2 and OCN mRNA levels in PDLSCs restores osteogenic differentiation in PDLSCs	[116]
PLA/CS	PLA/CS nanofiber	/	Promoted proliferation and osteogenic differentiation of BMSCs and upregulated the expression level of osteogenic genes	[121]
PLA/CA	PLA/CA nanofiber	/	Increased expression levels of cell mineralization genes and formation of mineralized junctions BMSCs, promoting osteogenic differentiation	[122]
M2-Exos	Exosomes	/	Increased expression levels of osteogenesis in BMSCs while restrained expression levels of osteoclast formation in BMDM	[125]
AMG-487 NP	Liposomal	AMG-487 NP	Reduced the number of osteoblasts and inhibits alveolar bone loss	[129]
Fibrin-ACP	Chitosan nanoparticles	ε-aminohexanoic acid	Promoted the differentiation of cementoblasts	[134]

#### Promoting periodontal membrane stem cell differentiation

**Fig. 6:**
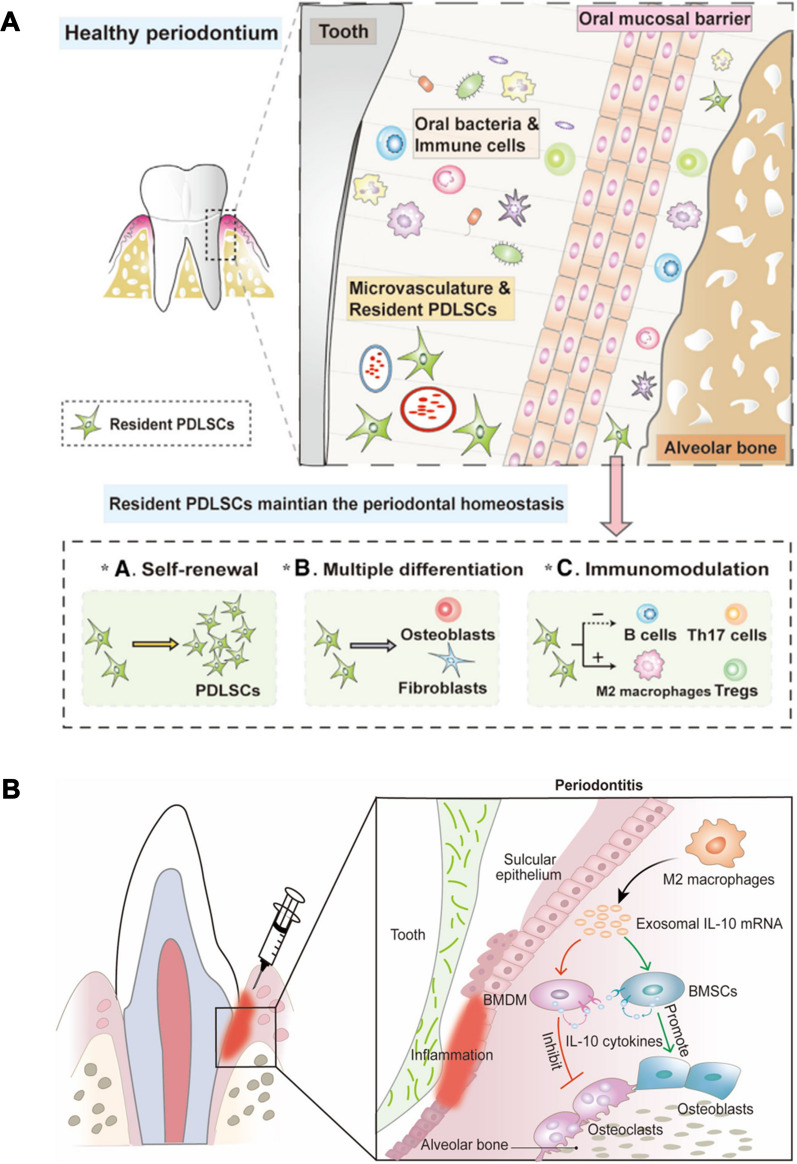
**A** PDLSCs through self-renewal, differentiating into osteoblasts and fibroblasts and regulating the host immune response to maintain the periodontal homeostasis. Reprinted with permission from Ref. [34] Copyright Oxford University Press. **B** Schematic illustration of M2-Exos promoting osteogenic differentiation of bone marrow stromal cells and inhibiting osteoclast formation of bone marrow-derived macrophages. Reprinted with permission from Ref. [125] Copyright Springer Nature

Periodontal membrane stem cells (PDLSCs), a subpopulation of mesenchymal stem cells, have self-renewal and immunomodulatory properties [108]. Moreover, PDLSCs can specifically repair the damaged periodontium [109]. PDLSCs can differentiate into fibroblasts, osteoblast-like cells and dental osteoclast-like cells to generate connective and dental osteoid tissue by nanomedicine (Fig. 6A) [110]. Therefore, regulating the differentiation of PDLSCs is a promising strategy for periodontium repair.

AuNPs promote the proliferation of human periodontal membrane stem cells via the classical Wnt/β-linked protein signaling pathway [111]. On this basis, Zhang et al. further investigated the potential of AuNPs to promote osteogenic differentiation of PDLCs [112]. AuNPs were prepared by chemical reduction using tetrachloroauric acid as the raw material. Next, L/D-cystine was modified on the surface of AuNPs. The results showed that L-cystine was more favorable AuNPs uptake than D-cystine. L-Cys-AuNPs promoted the proliferation of human PDLCs and upregulated the levels of the osteogenic genes ALP, collagen type I (COL-1), osteocalcin (OCN) and Runt-related transcription factor 2 (RUNX2). Pharmacological activation of autophagy was also significantly increased via osteoblast-differentiation activity. The results showed that the expression of autophagy-related genes and the levels of LC3 and SQSTM1 were increased in human PDLSCs cells after L-Cys-AuNPs treatment. A large amount of newly-formed alveolar bones and newly-formed periodontal ligaments were observed in a periodontal-defect rat model after L-Cys-AuNP-treatment. The microcomputed tomography (micro-CT) results of BV, bone volume/tissue volume (BV/TV), trabecular number (Tb.N), and Tb.Th indicated that L-Cys-AuNPs had more favour bone regeneration capacity than D-Cys-AuNPs. This study provides a new approach for the treatment of periodontitis with chiral-modified nanoparticles.

Liu et al. developed calcium fluoride nanoparticles (Nano-CaF_2_) for the osteogenic differentiation of PDLSCs. Calcium ions (Ca^2+^) are important throughout the cycle of bone formation to maturation and have the potential to contribute to bone differentiation [113]. Fluoride ions can inhibit tooth damage and play an important role in the suppression of dental caries by decreasing demineralization and increasing of remineralization. Nano-CaF_2_ with an average particle size of 53 nm was prepared by a spray dryer. The flexural strength, elastic modulus and hardness of Nano-CaF_2_ were 160 ± 10 MPa, 11.0 ± 0.5 GPa and 0.58 ± 0.03 GPa, respectively, which exceeded those of a commercial dental composite. The ALP activity after Nano-CaF_2_ treatment of human PDLSCs was 57, 78 and 55-fold higher than that of the control group on days 7, 14 and 21, respectively. Nano-CaF_2_ promoted the osteogenic and cementogenic induction of human PDLSCs by upregulating the expression of cementum adherence protein, cementum protein 1 and bone sialoprotein. Furthermore, Nano-CaF_2_ upregulated the osteogenic gene expression of RUNX2 and COL-1 in human PDLSCs.

Exosomes derived from healthy PDLSCs are potential components of osteogenic differentiation [114, 115]. Lei et al. isolated h-PDLSCs-exosomes from periodontal ligament tissue derived from healthy donors [116]. The diameter of h-PDLSCs-exosomes was 121.4 nm. h-PDLSCs-exosomes upregulated the mRNA levels of RUNX2 and OCN in the inflammatory periodontal ligaments of patients with periodontitis and promoted the expression of OCN osteogenesis-related proteins. Mechanistic studies found that h-PDLSCs-exosomes promote osteogenic differentiation by intervening Wnt signaling pathway. The mRNA levels of Wnt1, Wnt3a, Wnt10a, and β-Catenin were decreased in the inflamed periodontal ligaments of periodontitis patients after the treatment of h-PDLSCs-exosomes. However, the report indicated that the inflammatory environment leads to overactivation of Wnt signaling in PDLSCs, which inhibits the differentiation of PDLSCs in periodontitis [117]. Based on report, they believe that the contradictory results might be attributable mainly to the different sources and different culture conditions of PDLSCs. These results suggested that exosomes derived from PDLSCs can rescue the osteogenic capacity of PDLSCs, providing a new strategy for the treatment of alveolar bone loss in periodontitis.

#### Promoting bone marrow stem cell differentiation therapy

Bone marrow stem cells (BMSCs), which can be isolated and expanded in vitro, show differentiation capacity and have received widespread attention in periodontium regeneration [118, 119]. The local injection of BMSCs reverse receptor activator of NF-κB ligand/Osteoprotegerin (RANKL/OPG) expression contributed to the regeneration of periodontium in periodontitis mice [120]. A nanodelivery system consisting of chitosan nanoparticles incorporated into polylactic acid nanofibers (CS/PLA nanofibers) promoted the osteogenic differentiation of BMSCs and enhanced extracellular matrix mineralization [121]. The addition of chitosan nanoparticles increased the Young’s modulus and the stress and strain at break of the composites of fibers to resist masticatory forces while maintaining regenerative space. The scaffold architecture of CS/PLA nanofibers provides topographic cues to adherent cells, leading to cells along the axes of the architecture. The ability to control cellular alignment on scaffolds is advantageous for tissue regeneration in a specialized direction, such as the periodontal ligament. The result of alizarin red staining suggested that CS/PLA nanofibers could induce BMSCs to form mineralized nodules. CS/PLA nanofibers upregulated the mRNA expression levels of osteoblast-related factors, such as RUNX2, OPG, and RANKL. In another study, PLA/calcium alginate (PLA/CA) nanofibers produced similar results as to CS/PLA nanofibers by promoting the bone differentiation of BMSCs [122]. It can be concluded that combining polysaccharide composites with PLA nanofibers to promote BMSCs differentiation is a potential periodontitis treatment strategy.

#### Disturbing osteoclastogenesis

An important feature of periodontitis is alveolar bone resorption, which is exacerbated by the formation of osteoclasts [123]. The balance between osteoclasts and osteoblasts also influences the bone remodeling process [124]. Therefore, inhibiting osteoclast formation is important for alveolar bone regeneration and bone homeostasis in the periodontium.

Chen et al. extracted restorative M2-like macrophage exosomes (M2-Exos) to disrupt osteoclastogenesis in periodontitis treatment (Fig. 6B) [125]. M2-Exos had a round vesicle morphology with a double lipid membrane and their diameter was 30–150 nm. M2-Exos inhibited the expression of osteoclastogenic-related genes in bone marrow-derived macrophages (BMDMs). The average distance from the alveolar bone crest (ABC) to the cement-enamel junction (CEJ) was reduced significantly in periodontitis mice after M2-Exos treatment. In addition, the immunofluorescence image showed that tartrate resistant acid phosphatase (TRAP) expression was decreased in the periodontal tissues, which indicated the number of osteoclasts. IL-10 is considered a potential mediator of bone homeostasis in periodontitis. IL-10 can promote bone formation of BMSCs and inhibit osteoclast formation of BMDMs. Further mechanistic studies showed that M2-Exos upregulated the expression of IL-10 and inhibited osteoclast formation by delivering IL-10 mRNA to BMDMs, respectively. Although M2-Exos regulation of IL-10 provides a new target for tissue repair, it still has some limitations. For example, the ability of macrophages to yield exosomes is insufficient for clinical translation. Therefore, further studies on yield promotion are still needed, for example the production of exosomes secreted by cells can be increased by 3D culture or other techniques.

LPS-induced periodontal bone damage can be reduced by deleting the C-X-C motif chemokine ligand (CXCL) 9 and CXCL 10 receptors or by blocking the C-X-C motif chemokine receptor 3 (CXCR3) receptor with systemic administration of a CXCR3 antagonist [126, 127]. Sarah Hiyari and colleagues incorporated AMG-487 NPs (CXCR3 antagonist nanoparticles) into liposome nanoparticles self-assembled from palmitic acid and cholesterol to disturb ostecoclastogenesis [128, 129]. TRAP staining results showed that ostecoclastogenesis was inhibited after AMG-487 NPs-loaded liposome treatment. Micro-CT reconstruction analysis confirmed a 27.8% reduction in the level of bone loss after 1 week of administration. CXCR3 blockers may be a potential target for the treatment of bone loss by inhabiting and inhibiting ostecoclastogenesis in periodontitis treatment.

#### Accelerating cementoblast differentiation

The cementum is a layer of hard connective tissue covering the surface of compressed roots, consisting of cells and mineralized intercellular matrix, and is an important structure for maintaining the connection between the tooth and periodontium [130]. The major role of cementum is to serve as the site of attachment for principal collagen fibers (Sharpey’s fibers) [131]. Although various biological or engineering approaches have been attempted for periodontium neogenesis in preclinical and clinical applications, cementum regeneration remains a challenge in periodontitis due to the absence of blood vessels and matrix cells. Research has reported that enhancing the function of cementoblasts can efficiently promote cementum formation because cementoblasts can migrate to the impaired tooth root and form new cementum [132, 133]. Matrix proteases leading to fibrin degradation and apoptosis of OCCM30 cementoblasts. The protease inhibitor of ε-aminohexanoic acid can reverse this phenomenon [134]. Based on these findings, Chan Ho Parka et al. used fibrin incorporated with chitosan nanoparticles loaded with ε-aminohexanoic acid (Fibrin-ACP). SEM image showed that Fibrin-ACP with diameters of 30–50 nm produced a significant delay in fibrin degradation in a concentration dependent manner. Fibrin-ACP promoted the differentiation of cementoblasts in vitro, which was confirmed by the expression of cementoblast maturation- and biomineralization-related markers of RUNX2, osteocalcin, and bone sialoprotein. Fibrin-ACP strikingly increased periostin expression levels in PDL interfaces and Sharpey’s fiber insertions on mineralized tissue surfaces in vivo. Fibrin-ACP is inserted through Sharpey fibers to form the structural integration of the cementum-periosteum ligament-bone complex. The micro-CT images showed that Fibrin-ACP can induce 62.09 ± 10.47% bone regeneration to cover the tooth-root surface in the created defect site. Meanwhile, Fibrin-ACP promoted alveolar bone regeneration by increasing bone volume and root coverage of forked defect tops. In addition, Fibrin-ACP statistically enhanced tissue integration with fibrous connective tissue and cementum to develop tooth-supportive structures. The above results indicated that Fibrin-ACP exhibits powerful periodontal tissue repair potential by accelerating cementum regeneration.

### Synergistic nanotherapeutic strategies

With the continuous exploration of the pathogenesis of periodontitis, the close relationship between plaque, host immune response and periodontal tissue damage is being elucidated through multiple perspectives. In addition, periodontitis is a multifactorial outcome, and studies have shown that antimicrobial or immunomodulatory strategies alone are not sufficient to completely control the progression of periodontitis. Researchers need to explore synergistic nanotherapeutic strategies to improve the comprehensive treatment effect of periodontitis. The current synergistic treatment strategies with nanoparticles for periodontitis are summarized in Table 4.

**Table 4:** Synergistic nanotherapeutic strategies for periodontitis treatment

Nanoparticles	Delivery systems	Drugs	Outcome	References
*Antibacterial and immunomodulatory therapy*				
MPB-BA	MOF	Baicalein	Up-regulated of antioxidant genes (SOD-1, CAT and HO-1) expression to scavenge ROS and down-regulation of anti-inflammatory factors (TGF-β, IL-10)	[15]
AuAg@PC-Fe	AuAg nanoparticles	/	Photothermal antimicrobial and immunomodulatory treatment periodontitis	[136]
MZ@PNM	Macrophage membrane coating nanoparticles	Metronidazole	Interfered with the binding of Pg to macrophages and preventing Pg subversion of periodontal host immune response	[137]
*Antibacterial and periodontium regeneration therapy*				
PPZF-JNF	Nanofibers	ZIF-8 NPs and FK506	Zn^2+^ ions that interact with the bacterial surface to induce cellular deformation and lysis; up-regulation of the expression of osteogenic-related genes RUNX2, ALP, OCN	[140]
Gel MA-Z	Hydrogel	ZIF-8	Inhibited the growth curve of *Staphylococcus aureus* and increased the expression levels of the osteogenic genes (RUNX2, ALP, OCN, COL-1)	[143]
CTP-SA	Polymeric delivery system	/	ROS can be produced under blue light irradiation to exert antibacterial effects; appropriate Cu^2+^ facilitates the proliferation and osteogenic differentiation of BMSCs	[146]
*Immunomodulatory and periodontium regeneration therapy*				
AuNPs	Au nanoparticles	/	Enhanced expression of M2 markers; reduced RANKL/OPG ratio and promoted osteogenic differentiation	[152]
PssL-NAC NPs	Polymeric nanoparticles	NAC	Reduced LPS-induced apoptosis rate and osteoclast activity	[153]
TNPs	Treg cell membrane	poly (lactic-co-glycolic acid) nanoparticulate	Targeted CD80^+^/CD86^+^ on macrophages to inhibit osteoclastogenesis	[154]
*Trimodal synergistic therapy*				
CeO_2_@Ce6 NPs	Polymeric nanoparticles	Ce6	Regulated of macrophage polarization to improve microenvironment and increase gene expression levels of osteogenic markers	[155]
DPPLM-NPs	Polymeric delivery system	Minocycline Alpha lipoic acid	Reduced the level of intracellular ROS and promote the expression of osteogenic genes, reduce alveolar bone loss	[12]

#### Antibacterial and immunomodulatory therapy

Plaque biofilm is the initial factor in periodontitis, but the host immune response induced by plaque biofilm is the main reason for the aggravation of periodontitis [135]. After pathogenic microorganisms colonize the tooth surface, the balance between the pathogenic microorganisms and the host immune system will be broken, and the whole microbial community becomes pathogenic. In this case, the host immune response is also overactivated accompanied by the release of a variety of inflammatory factors and ROS. Inflammatory cytokines and ROS can eliminate bacteria, but further aggravate the inflammation of periodontal tissue. To address this pathological problem, the development of nanotherapeutic agents that can synergize antibacterial activity and immune regulation is an effective strategy for the treatment of periodontitis.

**Fig. 7:**
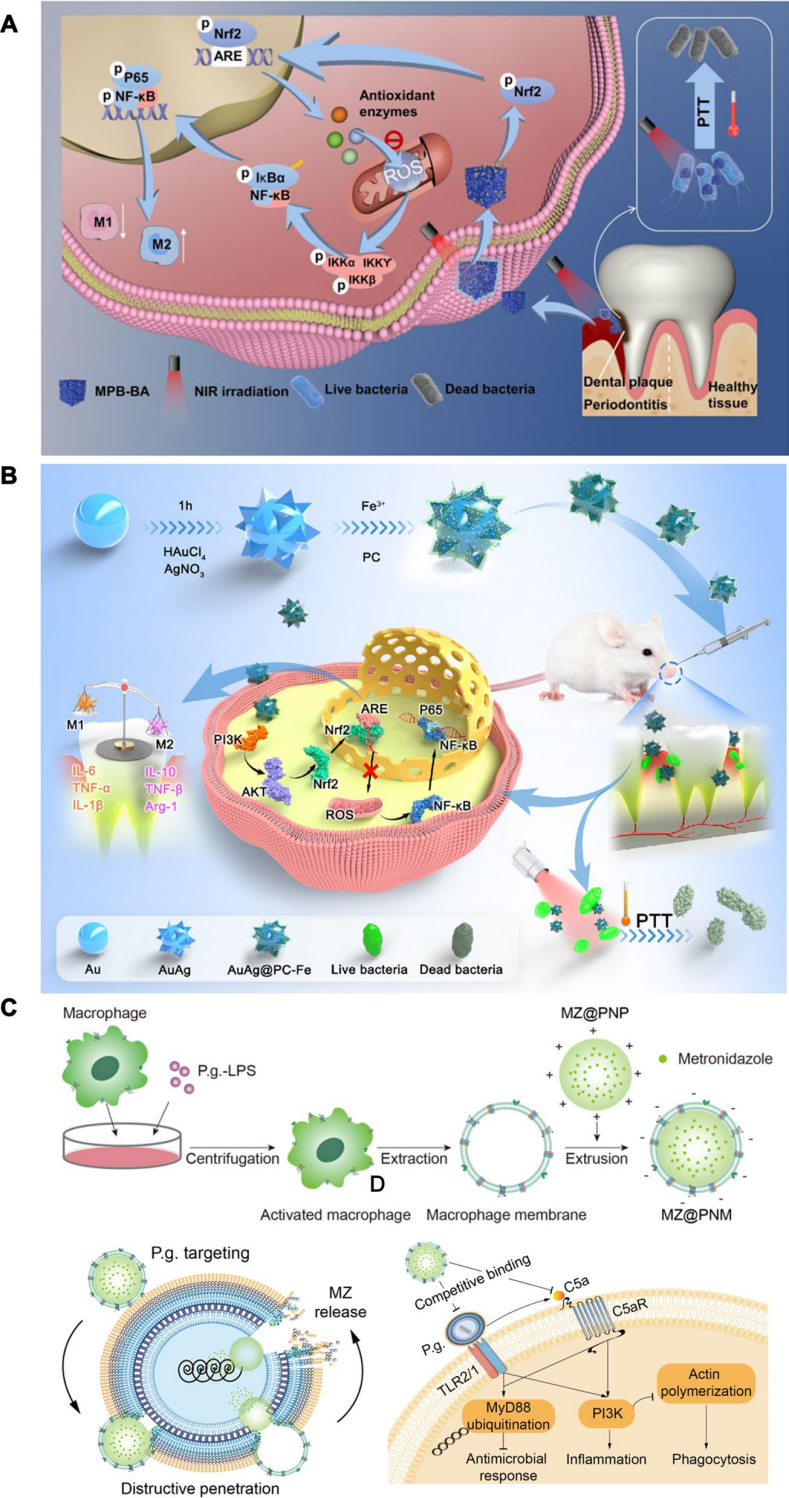
Schematic illustration of periodontitis treatment by antibacterial and immunomodulatory therapy. **A** Schematic illustration of antioxidant, anti-inflammatory mechanism and antibacterial effects of MPB-BA. Reprinted with permission from Ref. [15] Copyright KeAi. **B** Schematic illustration of antibacterial, antioxidant and anti-inflammatory effects of AuAg@PC-Fe. Reprinted with permission from Ref. [136] Copyright Elsevier. **C** Schematic illustration of the MZ@PNM for periodontitis treatment. Reprinted with permission from Ref. [137] Copyright American Chemical Society

Wu et al. developed an antibacterial and immunomodulatory synergistic nanotherapeutic strategy using mesoporous Prussian blue nanoparticles loaded with baicalein (MPB-BA) via photothermal therapy (Fig. 7A) [15]. MPB-BA exerted photothermal antibacterial effects under NIR, and released baicalein to modulate the macrophage phenotype. MPB-BA had a uniform size cube with an average diameter of 133.7 nm. MPB-BA exhibited a significant power—dependent increase with an 808 nm NIR laser. In addition, MPB-BA displayed excellent photothermal reversibility and cycling stability of MPB-BA, and the photothermal conversion efficiency of MPB-BA was 27.6%. MPB-BA was able to disrupt the integrity of Pg and Fn under NIR. In addition, MPB-BA reduced 6.8-fold ROS compared with the control group, and increased the expression of the antioxidant genes SOD-1, CAT, NQO-1 and HO-1 in macrophages. A phenotypic shift in macrophages from M1 to M2 was also observed. Mechanistic studies showed that MPB-BA regulated macrophage polarization by promoting Nrf2 phosphorylation and nuclear translocation.

Based on the photothermal antimicrobial and immunomodulatory synergistic treatment strategy, Wang and his colleague designed metal-phenolic networks (MPNs) loaded with AuAg nanoparticles (AuAg@PC-Fe) (Fig. 7B) [136]. MPNs are formed by the coordination of metal ions and polyphenols. Fe^3+^ and procyanidins form a stable PC-Fe film coated with AuAg NPs, which enhances photothermal properties. The average thickness of AuAg@PC-Fe was 10.61 nm, and the zeta potential of AuAg@PC-Fe decreased from − 13.3 to − 25.6 mV after the AuAg core bonded with the PC-Fe film. AuAg@PC-Fe reached 50 °C after irradiation with 808 nm NIR, and the photothermal conversion efficiency of AuAg@PC-Fe was 53.3%, which suggested that AuAg@PC-Fe exhibited efficient photothermal properties. The antibacterial ability of AuAg@PC-Fe was stronger than that of AuAg due to certain antibacterial properties of the PC-Fe coating. The colony forming units of the Fn and Pg biofilms decreased by seven orders of magnitude after AuAg@PC-Fe was irradiated with NIR light treated. In addition, the level of ROS decreased eightfold in macrophages after AuAg@PC-Fe treatment. AuAg@PC-Fe inhibited the expression of CD86 and promoted the expression of CD206 in macrophages which suggested that AuAg@PC-Fe effectively induced the transformation of M1 macrophages into M2 macrophages. Mechanistic studies showed that AuAg@PC-Fe promoted Nrf2 phosphorylation through the activation of the PI3K/Akt signaling pathway, eliminated ROS, and inhibited the nuclear translocation of P65 in the NF-κB signaling pathway to regulate the immune response.

In addition to the direct elimination of pathogenic bacteria, intervention in the interaction between bacteria and immune cells is a feasible synergistic strategy. Pg inhibits the bactericidal capacity and phagocytosis of macrophages through TLR2/1 and complement component 5a receptor (C5aR)-dependent signaling pathways. Based on this, Yan et al. prepared metronidazole-loaded nanoparticles with a macrophage membrane coating (MZ@PNM) to target Pg and restore the antimicrobial functions of local immune cells (Fig. 7C) [137]. TEM images showed that the size of MZ@PNM ranged from ~ 214 to ~ 247 nm and the ζ-potential ranged from + 40 to − 26 mV after the cell membrane was successfully coated. MZ@PNM penetrated the bacterial membrane of Pg via membrane fusion, and cationic nanoparticles containing metronidazole were released. Cationic metronidazole nanoparticles had a strong destructive effect on the bacterial membrane. In addition, MZ@PNM competitively binds to Pg, and interferes with the binding of Pg to macrophages. MZ@PNM prevents Pg from activating an excessive host immune response by neutralizing C5a on the Pg surface via C5aR. The protein expression of PI3K was inhibited and that of MyD88 was increased in macrophages after the addition of MZ@PNM. These results indicate that cutting off the link between pathogenic bacteria and immune cells is a strategy worth exploring for synergistic antibacterial and immunomodulatory effects.

#### Antibacterial and periodontium regeneration therapy

Lipopolysaccharides on bacterial biofilms are highly toxic to the periodontium, inhibiting the growth of fibroblasts and activating osteoclast activity. Collagenases produced by the pathogen destroy periodontal connective tissue, leading to attachment loss and bone collagen degradation. Therefore, it is possible to enhance the therapeutic effect of periodontitis by combining anti-bacteria and periodontium regeneration strategies. In recent years, some new antibacterial nanomaterials, such as Zn nanoparticles, Cu_2_O nanoparticles and nanofibers, have been introduced into the combined treatment strategy for antibacterial and periodontium regeneration.

**Fig. 8:**
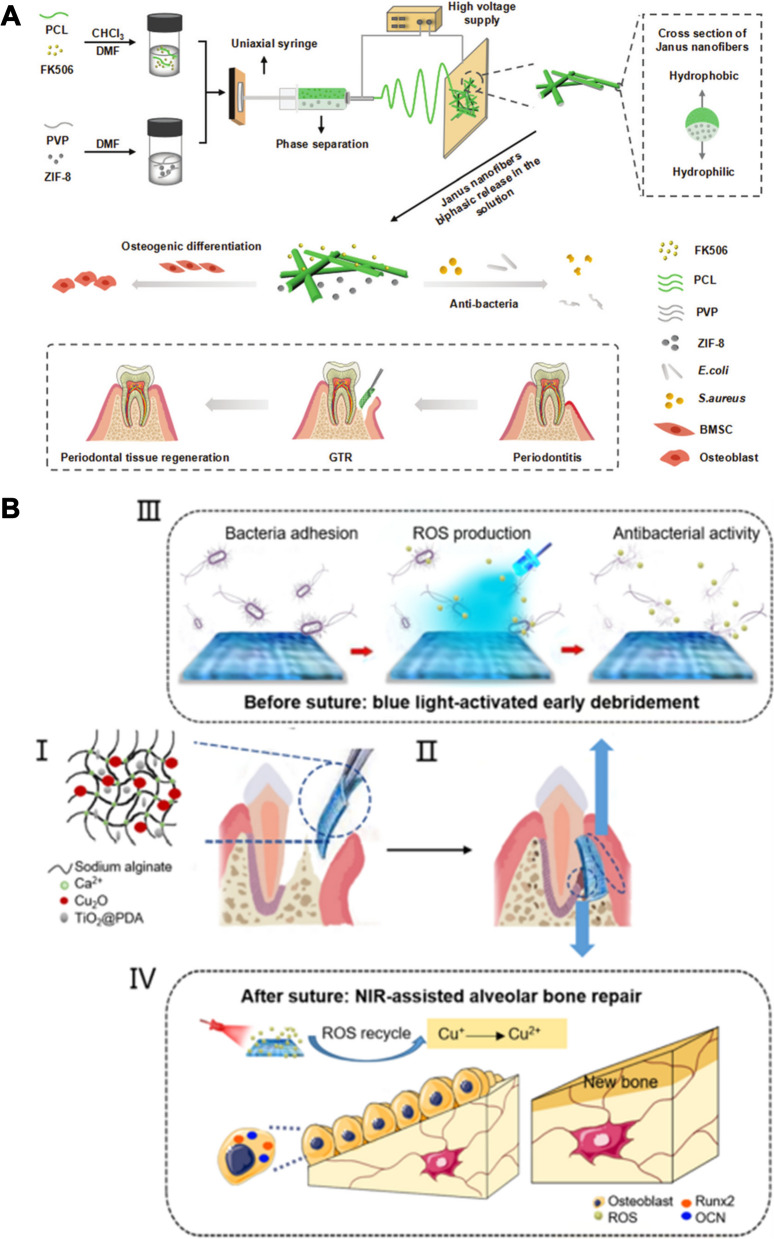
Schematic illustration of periodontitis treatment by antibacterial and immunomodulatory therapy. **A** Schematic illustration for the preparation and application of PPZF Janus nanofibers. Reprinted with permission from Ref. [140] Copyright Royal Society of Chemistry. **B** Schematic illustration of the construction of CTP-SA and antibacterial and immunomodulatory of CTP-SA under blue + NIR. Reprinted with permission from Ref. [146] Copyright American Chemical Society

Zeolite imidazole skeleton-8 (ZIF-8) consists of zinc ions and 2-methylimidazole. ZIF-8 releases Zn^2+^ and 2-methylimidazole to exert antimicrobial properties in the acidic microenvironment of bacterial infections. In a series of recent studies, ZIF-8 nanoparticles have been shown to be effective nanodrug or nanocarrier for antimicrobial therapy and tissue regeneration [138, 139]. Sun et al. developed a multifunctional drug delivery system (PPZF JNF) that exerts active osteogenic effects while preventing bacterial infection (Fig. 8A) [140]. In their study, ZIF-8 nanoparticles and FK506 were preserved in nanofibers. Nanofibers were prepared via the electrostatic spinning method by polycaprolactone and polyvinylpyrrolidone. The TEM images confirmed that the ZIF-8 NPs and FK506 were distributed in different spaces of the nanofibers. The number of *Escherichia coli* and *Staphylococcus aureus* were significantly reduced after treatment with PPZF JNF. Benefiting from by the excellent antibacterial effect of PPZF JNF, the osteogenic activity was enhanced in the periodontium. The expression levels of osteogenic genes (RUNX2, ALP, OCN) were increased in BMSCs. The distance of the CEJ-ABC contracted 21.8% in periodontitis rats after PPZF JNF implantation. In addition, BV/TV increased by 25.7% and Tb.N increased by 34.5%. Trabecular separation (Tb.sp) was reduced by 0.16-fold. The metal nano-antibacterial agents of ZIF-8 play an important role in periodontal bone tissue repair via antibacterial effects.

In addition to indirect effects, ZIF-8 can enhance osteoblast activity through the local release of Zn^+^ ions to reach the effective threshold. Zn^2+^ in a suitable concentration range activates the secretion of various growth factors, such as epidermal growth factor, insulin-like growth factor-1, vascular endothelium growth factors [141]. ZIF-8 encased in a polycaprolactone/collagen membrane can markedly enhance osteogenesis in vitro and in vivo [142]. In another study, Liu and his colleagues constructed an injectable photo polymeric composite hydrogel (Gel MA-Z) by loading ZIF-8 nanoparticles into gelatine methacrylate [143]. ZIF-8 NPs were first synthesized via a hydrothermal method and then dispersed in gelatine methacrylate solution to prepare Gel MA-Z. As a smart photosensitive gel, Gel MA-Z could be noninvasively injected into the irregular periodontal pocket and transformed into a gel state under UV light. The methacrylate gelatine substrate increased the retention of ZIF-8 in the periodontium and reduced the hydrolysis of ZIF-8 NPs to exert a protective effect. The expression of osteogenic genes (ALP, RUNX2, COL-1, OCN) was increased after co-culture with Gel MA-Z. The growth curve of *Staphylococcus aureus* was inhibited by Gel MA-Z and the results of the live/dead bacterial staining kit were consistent with this result. In the periodontitis rat model given Gel MA-Z, the micro-CT results showed that the CEJ-ABC distance was closer to that of the blank group. In conclusion, ZIF-8 nanoparticles have broad application prospects in antibacterial and periodontium regeneration synergistic therapy.

Au nanomaterials have been widely used in antimicrobial therapy because of their unique optical properties and high biocompatibility. Dong et al. designed a hydrogel modified by Au nanoparticles with epigallocatechin gallate (E-Au@H) to achieve bactericidal and periodontium regeneration synergistic therapy [144]. E-Au@H was capable of providing stable photothermal effects for photothermal therapy. E-Au@H increased the temperature to 50.7 °C with 808 nm NIR laser. The antibacterial rates of E-Au@H against *Escherichia coli* and *Staphylococcus aureus* were 92% and 94%, respectively. SEM showed that the shape of the *Staphylococcus aureus* cell membrane was greatly damaged and that the surface collapsed obviously after treatment of E-Au@H with NIR laser irradiation. Furthermore, the activity of ALP was increased fivefold, and the number of calcified nodules was increased threefold in BMSCs after E-Au@H treatment with NIR. The mRNA expression levels of RUNX2, ALP and OCN were all increased in BMSCs, which may be due to the continuous release of epigallocatechin gallate triggered by E-Au@H through NIR light. Compared with the E-Au@H group, the ABC-CEJ distance in the E-Au@H group irradiated by NIR was smaller, and the collagen fibers were more neatly arranged and dense. These results showed that the Au nanomaterials exhibited antibacterial and bone tissue repair-promoting abilities under NIR light irradiation.

ROS cycling-induced copper Cu ion (Cu^2+^) oxidation is a promising strategy for combined antibacterial and periodontium regeneration [145]. Xu et al. proposed an injectable sodium alginate hydrogel composite (CTP-SA) composed of cubic cuprous oxide (Cu_2_O) and polydopamine coated titanium dioxide (TiO_2_@PDA) nanoparticles (Fig. 8B) [146]. TiO_2_@PDA could produce ROS under blue light irradiation, inducing the oxidation of Cu^+^ to Cu^2+^, and Cu^2+^ was beneficial to bone mesenchymal stem cell proliferation and osteogenic differentiation. The antibacterial effect of CTP-SA against *Staphylococcus aureus*, *Escherichia coli* and *Streptococcus mutans* was increased to 97.73 ± 0.81%, 98.52 ± 0.78% and 96.37 ± 2.99%, respectively under blue light. The fewest colonies were observed in the periodontitis rats after CTP-SA + blue light treatment. The temperature of CTP-SA increased to 48.5 ± 0.71 °C within 30 min under NIR irradiation, which is a suitable value for osteogenesis. The micro-CT results showed that the CTP-SA + blue light/NIR treated group showed the shortest distance between the bone crest and CEJ, which also confirmed that the delivery system promoted alveolar bone repair.

#### Immunomodulatory and periodontium regeneration therapy

As mentioned earlier, some immune cells (M1 macrophages, Th1 cells, Th17 cells), proinflammatory cytokines, ROS, chemokines, and other immune mediators can directly or indirectly contribute to the destruction of the periodontium. For example, Th17 cells induce the production of IL-17, which in turn upregulates the mRNA expression of MMP-1 and MMP-3 in the periodontium [147]. This leads to collagen and proteoglycan degradation, ultimately promoting periodontal tissue destruction [148]. M1 macrophages release proinflammatory cytokines, such as TNF-α, IL-1β, IL-6, IL-12, and IL-23 [149]. TNF-downregulates bone matrix and RUNX2 expression in osteoblasts and upregulates RANKL expression in osteoclast precursors and osteoblasts, ultimately leading to periodontium destruction [37]. IL-1β promotes collagenase production in osteoblasts and increases collagen degradation in the periodontium [150]. IL-6 upregulates RANKL receptor activator expression in osteoblasts and induces osteoclast differentiation and alveolar bone resorption [151]. Therefore, the synergistic strategy of immunomodulation and periodontium regeneration by nanomedicines is expected to significantly improve the therapeutic effect of periodontitis.

In addition to excellent photothermal antibacterial properties, AuNPs can modulate macrophage phenotypes and attenuate osteoclast activity, making them a multifunctional therapeutic nanoparticle for periodontitis treatment [152]. AuNPs decreased the mRNA levels of M1-type macrophage-related factors (TNF-α, IL-6), and promoted M2-type macrophage-related factors (Arg-1, IL-10 and TGF-β). Notably, AuNPs-treated M2-type-macrophage conditioned medium promoted the osteogenic differentiation of human PDLSCs. The mRNA transcription levels of the osteogenic factors ALP and COL-l in human PDLSCs were all increased in AuNPs-modulated macrophage conditioned medium. Similarly, AuNPs-modulated macrophage-conditioned medium also facilitated the formation of mineralized nodules in human PDSLCs, as shown by Von Kossa staining. In addition, the intracellular RANKL/OPG ratio was reduced, which suggested weakened osteoclastogenic activity. H&E and Masson staining showed that the elastic fibers and collagenous fibers were denser and more well-organized in the periodontium after AuNP treatment. The micro-CT results further confirmed that AuNPs could promote alveolar bone repair by regulating macrophage function.

**Fig. 9:**
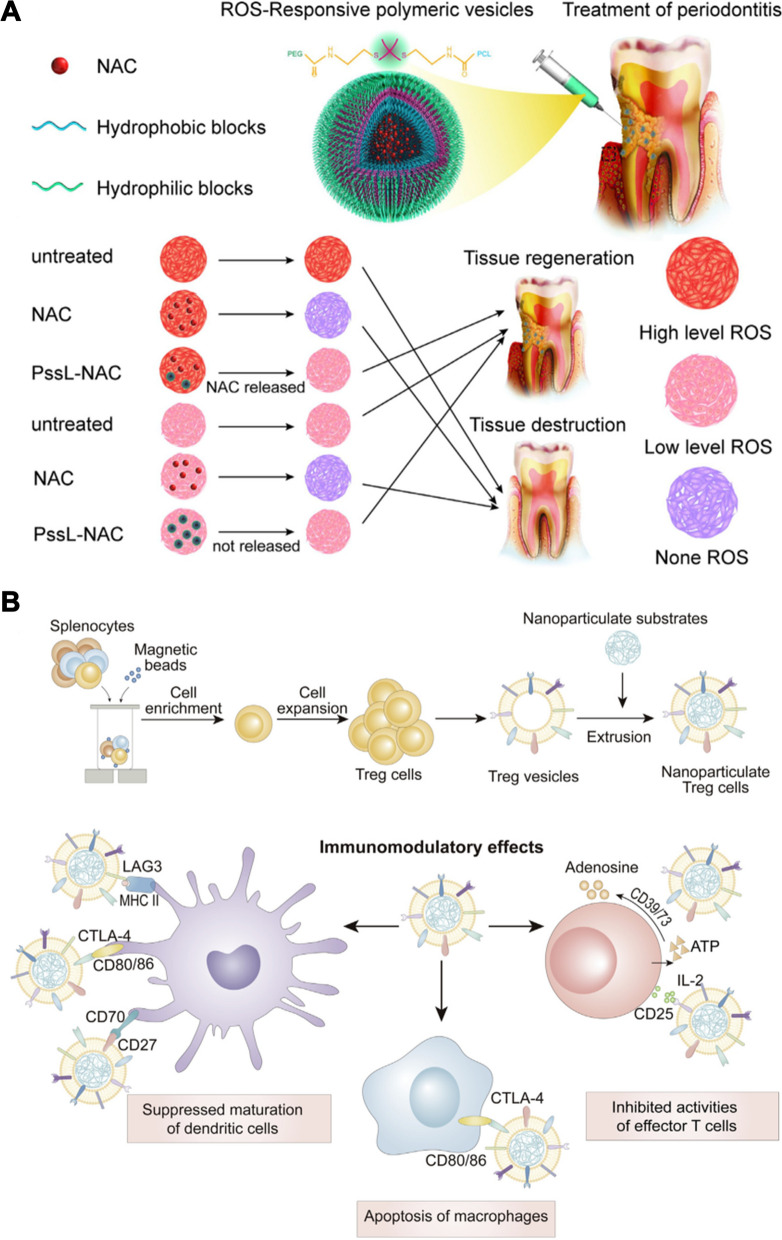
Schematic illustration of periodontitis treatment by immunomodulatory and periodontium regeneration. **A** Schematic of PssL-NAC NPs regulates ROS levels exerts different effects on tissue regeneration. Reprinted with permission from Ref. [153] Copyright Elsevier. **B** Schematic illustration of TNPs inhibit macrophage-osteoclast differentiation, suppress DCs maturation and regulatory effector T cells. Reprinted with permission from Ref. [154] Copyright Elsevier

The reduction in cytokines and ROS secreted by immune cells contributes to the osteogenic differentiation of PDLSCs. Based on this, tailor-made ROS-cleavable amphiphilic polymer nanoparticles for removing ROS (PssL-NAC NPs) were designed by N-acetylcysteine (NAC, a ROS scavenger) (Fig. 9A) [153]. Thioketal bonds of the PssL-NAC NPs structure respond to oxidative stress in microenvironments, and then NAC is released to regulate intracellular ROS levels. The PssL-NAC NPs had homogeneously spherical morphologies and the average diameter was ~ 155 nm. The late-stage and early apoptosis ratios were 24.1% and 28.2%, respectively in human PDLSCs as the concentration of LPS increased, which suggests the damaging effect from the high levels of inflammation and ROS. The apoptotic ratio of human PDLSCs was decreased after PssL-NAC NPs treatment due to the ROS scavenging activity of PssL-NAC NPs. In addition, the results showed that PssL-NAC NPs increased the mRNA expression of osteogenic markers (BMP-2, RUNX2). Alveolar bone height and bifurcation sites were restored in periodontitis rats after treatment with PssL-NAC NPs. Notably, low ROS levels may have promoted osteogenic differentiation of PDLSCs. Stimulation with 5 μg/ml LPS increased ALP activity, while stimulation with 10 μg/ml LPS decreased ALP activity in PDLSCs. These results suggest that maintaining ROS homeostasis in the periodontium, rather than eliminating ROS completely, is essential for periodontium regeneration.

Treg cell membrane-encapsulated poly (lactic-co-glycolic acid) nanoparticles (TNPs) retain the intrinsic membrane proteins of Treg cells, and can directly interact with overactive immune cells to inhibit macrophage-osteoclast differentiation (Fig. 9B) [154]. TEM images showed that the Treg membrane was completely wrapped around the poly (lactic-co-glycolic acid) nucleus with a core–shell morphology. TNPs target CD80/86 on macrophages to inhibit osteoclastogenesis. The downregulation of osteoclast-related genes, including nuclear factor of activated T cells 1 (NFATc-1) and RANKL, further supports the inhibitory effect of TNPs on osteoclast differentiation. Alveolar bone loss was inhibited in TNPs-treated periodontitis mice, and the expression of inflammation and osteoclast-related genes was significantly downregulated in gingival tissue sections. In addition, TNPs inhibit CD4^+^ T cell proliferation by affecting co-stimulatory molecule binding. Simultaneously, TNPs downregulate the expression of CD70, CD80 and CD86 on the surface of dendritic cells via Treg cell surface proteins, thereby further inhibiting the maturation of dendritic cells. These results confirmed the potential of biomimetic nanotechnology of the cell membrane to achieve immunomodulatory and periodontium regeneration synergistic therapy in periodontitis.

#### Trimodal synergistic therapy

At present, the treatment of periodontitis is no longer limited to a single strategy. Nanodelivery systems with trimodal synergistic therapy of antibacterial, immunomodulatory and periodontium regeneration are being explored.

**Fig. 10:**
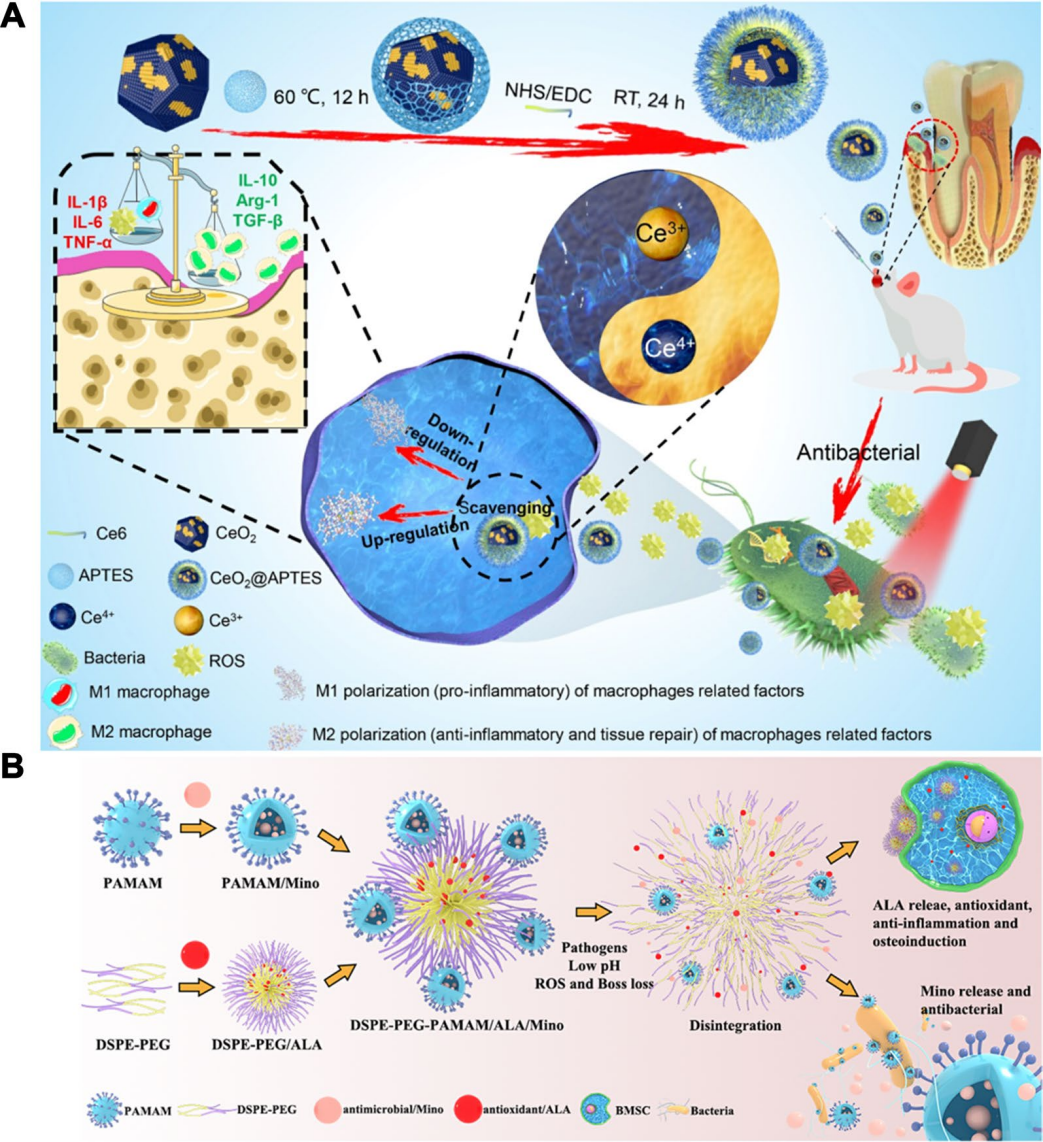
Schematic illustration of periodontitis treatment by trimodal synergistic therapy of antibacterial, immunomodulatory and periodontium regeneration. **A** Schematic illustration of CeO_2_@Ce6 in synthesis, the antibacterial mechanism and modulating the polarization of macrophages. Reprinted with permission from Ref. [155] Copyright Elsevier. **B** Schematic illustration of preparation of DPPLM NPs. Reprinted with permission from Ref. [12] Copyright KeAi

PDT produces large amounts of ROS to exert antimicrobial effects, but excessive ROS can cause immune imbalance and damage to the periodontium. Based on the correlation between the antibacterial effect of ROS and the immune regulation effect on periodontium regeneration, Dong et al. prepared a multifunctional nanodelivery system by modifying the photosensitizer Ce6 on the surface of CeO_2_ nanoparticles (CeO_2_@Ce6 NPs) (Fig. 10A) [155]. Pg and Fn biofilms were reduced by approximately 4 orders of magnitude after red light irradiation with CeO_2_@Ce6 NPs. CeO_2_@Ce6 NPs also significantly inhibit fimbriae and cysteine protease gene expression in biofilms. CeO_2_@Ce6 NPs inhibited bacterial proliferation through ROS, and then scavenging of residual ROS by CeO_2_. The decreased ROS promoted the transformation of macrophages from M1 to M2. Noticeably, the application of CeO_2_@Ce6 NPs to murine MC3T3-E1 cells (osteoblast progenitors) exhibited significant osteogenic potential. The mRNA expression of osteogenic-related factors (ALP, COL-1, RUNX-2) was increased in MC3T3-E1 cells after CeO_2_@Ce6 treatment.

Wang and his colleagues designed a dual pH- and enzyme-responsive nanodelivery system (DSPE-PEG-PAMAM/ALA/Mino) for trimodal synergistic therapy (Fig. 10B) [12]. DSPE-PEG-PAMAM/ALA/Mino consists of two main components: poly(aminoamine) (PAMAM) loaded with the antimicrobial agent minocycline hydrochloride and 1,2-distearoyl-sn-glycero-3-phosphoethanolamine-polyethylene glycol (DSPE-PEG) loaded with the antioxidant alpha lipoic acid (ALA). PAMAM is sensitive to pH and is used to induce drug release. DSPE-PEG, a PEGylated lipid polymer, is sensitive to bacterial enzymes. Therefore, DSPE-PEG-PAMAM/ALA/Mino could intelligently release minocycline hydrochloride and ALA under periodontitis pathologic conditions. Pharmacodynamic studies showed that only a small number of bacteria were scattered after DSPE-PEG-PAMAM/ALA/Mino treatment, showing positive antibacterial activity. DSPE-PEG-PAMAM/ALA/Mino inhibited the inflammatory process by reducing the overproduction of ROS and inducible nitric oxide synthase. A significant reduction in the CEJ-ABC distance was observed with a mean reduction of 0.357 mm after DSPE-PEG-PAMAM/ALA/Mino treatment. Mechanistic studies further indicated that DSPE-PEG-PAMAM/ALA/Mino upregulate the mRNA expression of ALP and OCN for BMSCs osteogenic differentiation. Overall, these smart-response nanosystems exhibit a trimodal role of antibacterial, immunomodulatory and periodontium repair in periodontitis treatment.

## Challenges and future prospects

Nanotherapeutic strategies have made breakthroughs in the treatment of periodontitis, such as enhanced drug solubility, sustained drug release, targeting of specific cells, and synergistic therapeutic effects. However, there are still some challenges and practical problems to be solved to further improve the efficacy and clinical translation of nanotherapeutic strategies in periodontitis.

### Elimination or balance?

There are up to approximately 700 species of microorganisms in the human oral cavity [156]. Teeth, gingival sulcus and oral mucosa provide habitats for the colonization of these microorganisms, forming a complex microbial ecological community. Pg is considered the keystone pathogen in the development of periodontitis [157]. In addition, *Aggregatibacter actinomycetemcomitans*, *Tannerella forsythia*, *Treponema denticola*, and *Filifactor alocis* are suspected in periodontitis [158]. Most current nano-antibacterial treatment strategies, such as spectral antibiotics or PDT, inhibit the development of periodontitis by eliminating all bacteria present in the periodontium. However, it has been shown that certain probiotics, such as *Lactobacillus* and *Streptococcus dentisani* can neutralize or hinder the driving factors of periodontal disease [159]. The potential mechanisms of *Lactobacillus* include the suppression of pathogens, the competitive inhibition of pathogenic bacteria attachments and combating bacterial biofilms [160]. Jung et al. showed that cell free supernatants prepared form *Lactobacillus curvatus* MG5246 decreased TNF-α, IL-6 and cyclooxygenase-2 gene expression in Pg-LPS-stimulated human gingival fibroblasts, which opens a window for new therapeutic strategies to prevent periodontitis [161]. *S. dentisani* inhibits the colonization of Pg and Fn in human gingival fibroblasts, and decreases the production of pro-inflammatory cytokines secreted by Pg and Fn [162]. Thus, we may be able to employ specific targeted nanosystems or biomimetic strategies to exert selective antimicrobial effects without harming beneficial bacteria and normal tissue cells. Restoring the homeostasis of the oral microbiota balance instead of eliminating all microorganisms is the future direction of antimicrobial therapy with nanodelivery systems.

In the microenvironment of periodontal pathology, overactive macrophages or some T cells produce a large amount of ROS, and the accumulation of ROS activates related inflammatory signaling pathways and osteoclast maturation, ultimately leading to periodontal tissue damage. Current nanotherapeutic strategies aim to eliminate ROS as the ultimate goal. However, several studies have found that low levels of ROS may protect the regenerative potential of stem cells [163]. This is because the low levels of intracellular ROS mediate the transient activation of c-Jun N-terminal protein kinase and aid cell survival by activating activator protein-1 transcription factors and anti-apoptotic genes [163, 164]. Therefore, we are considering whether ROS in the periodontium can be maintained at an acceptably low level to promote periodontium regeneration and play an antibacterial role without generating an immune response or oxidative stress.

### Opportunities of nano-targeted therapy for periodontitis

Most current nanotherapeutic strategies for periodontitis are non-targeted delivery systems, which cannot target specific cells or bacteria. It is possible to further improve the therapeutic effect of nanodrug delivery system through the precise regulation of key cells in the periodontium. Compared to healthy individuals, patients with periodontitis express higher levels of integrins, which is further upregulated in the advanced stages of periodontitis [165, 166]. The RGD peptide (arginine-glycine-aspartate) specifically binds to integrins and enhances the adhesion between stimulated cells and drug carriers, which can mediate nanoparticle adhesion to inflammatory epithelial cells and allow long-term retention of nanoparticles in periodontal pockets [167, 168]. Yao et al. synthesized an epithelial cell-targeted nanoparticle delivery system based on minocycline-loaded poly (ethylene glycol)–poly (lactic acid) nanoparticles, using coupled RGD peptide modification (RGD-NP-MIN) [169]. The uptake of RGD-NP-MIN was 3.1-fold that of unmodified nanoparticles. Cell attachment assays also showed that RGD-NP-MIN bound 5.0-fold more Calu-3 cells than unmodified nanoparticles. In vivo pharmacodynamic results showed significant improvements in the clinical periodontal parameters of the plaque index, gingival index and periodontal pocket depth. These results suggested that epithelial cell-targeted nanoparticles provide an effective therapeutic strategy for periodontitis. Recently, there have been reports on periodontitis treatment strategies targeting macrophages and Pg [170]. Our group developed the folate-modified genistein-loaded liposomes to target macrophages. Macrophages were transformed from M1 to M2 phenotype in periodontitis tissue. Folate-modified genistein-loaded liposome could regulate the TLR4/MyD88/NF-κB axis in macrophages and promote osteogenic differentiation of PDLSCs [171]. In addition, some other immune cells (T cells, B cells, neutrophils) and periodontal tissue cells (periodontal stem cells, gingival fibroblasts, etc.) may be potential targets for the targeted regulation of nanodrug delivery systems in periodontitis treatment.

### Opportunities of nanomedicines integrated with multi-platforms

The physiological functions of the oral cavity, such as saliva secretion, mastication, and vocalization, may lead to the quickly elimination of nanomedicines in the lesion area of periodontist. The combination of nanomedicines and delivery platforms can significantly improve the retention time of nanomedicines in the periodontal pocket and local drug concentration in the lesion area, which is a feasible direction for clinical translation.

At present, the nanomedicines were integrated into the “smart hydrogel” platforms for periodontist therapy. We previously systematically reviewed the development of smart hydrogel for periodontitis treatment [172]. The responsive groups in the smart hydrogel structure allow in-situ phase transition between the solution and solid state in the periodontal pocket. Li et al. prepared an injectable photosensitive hydrogel by doping dexamethasone-loaded ZIF-8 nanoparticles into a photo crosslinked matrix of polyphosphate methacrylate and GelMA for periodontitis treatment [173]. In addition, the smart hydrogel can control release nanomedicines through the stimuli-responses including ROS, pH, light, enzymes, etc. MZ@PNM was integrated with chitosan/sodiumβ-glycerophosphate system for pH-responsive release properties due to the remaining amino groups on chitosan matrix. The hydrogel structure was significantly collapse after incubation at pH 4.0 than at pH 7.4, which could release the nanoparticles in the acidic periodontal environment [137].

In addition, bio-sponges with porous structures have ideal loading properties, biocompatibility and biodegradability, which gain attention in the field of hemostasis and wound healing. Wang's group prepared bio-sponges based on carboxymethyl chitosan/ poly-gamma-glutamic acid/platelet-rich plasma that adhere and coagulate red blood cells to accelerate blood clotting via releasing epidermal growth factor and vascular endothelial growth factor [174]. In the field of periodontitis treatment, bio-sponge platforms are absorbable and relatively inert during bone regeneration. Mesenchymal stem cell exosome-loaded collagen sponge promoted newly-formed bone and periodontal ligament regeneration by increasing periodontal ligament cell migration and proliferation [175].

Electrets have attracted widespread interest in bone regeneration and drug delivery due to the great performance in endogenous electrical stimulation for enhancing cell proliferation and differentiation [176]. Yu et al. designed an electret-based host-coupled biological nanogenerator through electrical stimulation to increase cytoplasmic calcium ions to activate osteogenic differentiation. The result showed that electret significantly promoted the osteogenic differentiation of bone marrow mesenchymal stem cells in vitro and bone regeneration in vivo [177]. The development of nanocomposite delivery systems utilizing the advantages of charge retention and high surface charge density of electrets nano-materials such as ZnO and SiO_2_, may be a potential therapeutic strategy for the treatment of periodontitis.

### Opportunities of periodontitis comorbidities treatment

**Fig. 11:**
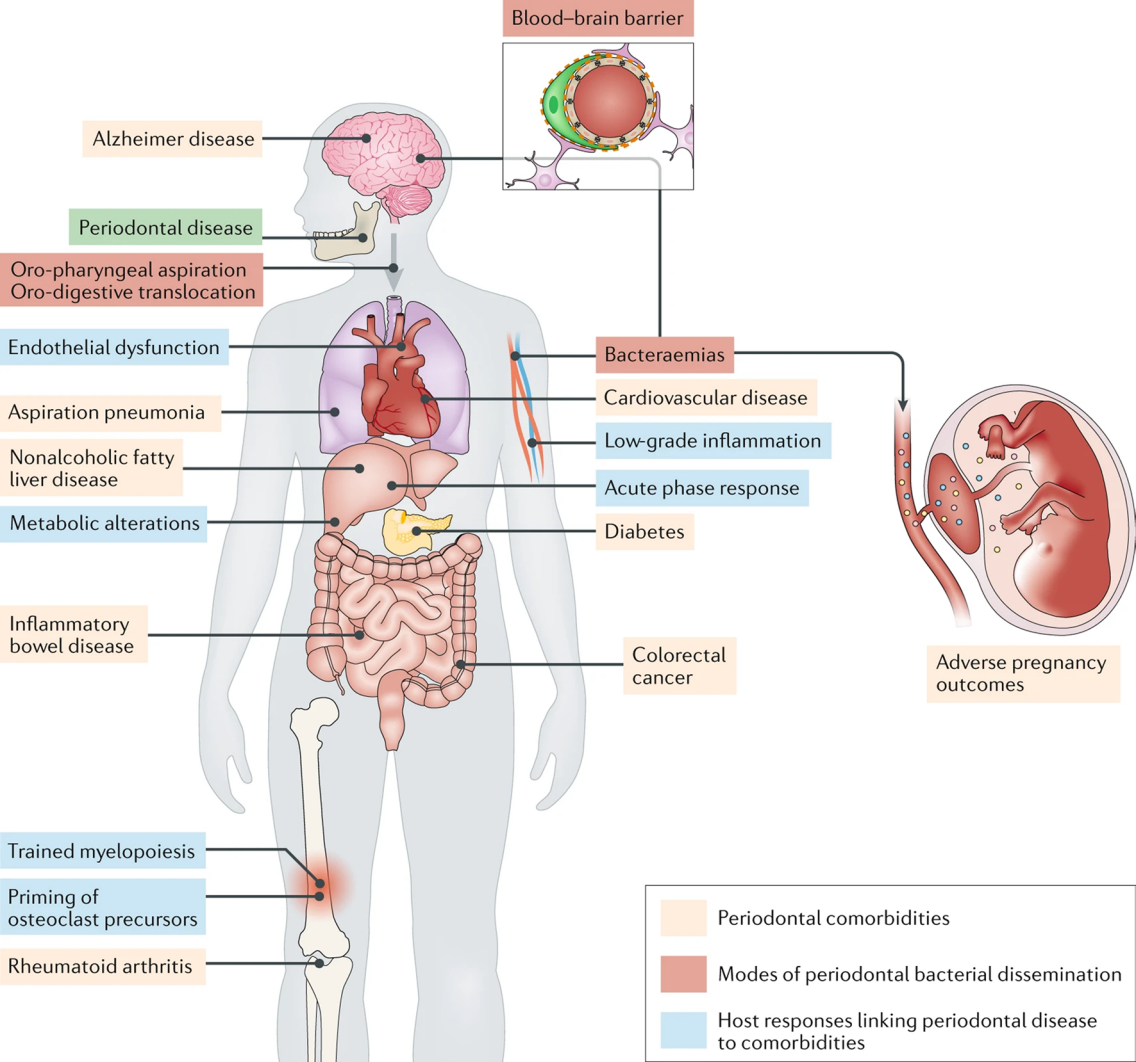
Periodontitis and associated inflammatory comorbidities. Reprinted with permission from Ref. [186] Copyright Springer Nature

Periodontitis is associated with diabetes, rheumatoid arthritis, Alzheimer's disease, hypertension, inflammatory bowel disease, and even autoimmune diseases and cancer (Fig. 11) [178–180]. Therefore, nanodelivery systems also have promising applications in the treatment of periodontitis with comorbidities. For instance, there is a bidirectional association between periodontitis and diabetes [181]. Periodontitis may increase the prevalence of diabetes and affect the effective control of blood glucose [182, 183]. On the other hand, metabolic disorders in diabetic patients lead to excessive production of ROS, which has a damaging effect on alveolar bone. The damage to periodontal tissue in diabetic patients with periodontitis was more destructive than that in patients with periodontitis alone. To address this problem, Zhao et al. developed a ROS-responsive drug delivery system loaded with both doxycycline and metformin that worked effectively in periodontitis with diabetes [184]. Wang et al. prepared injectable nano-hydrogels using mesoporous silica nanoparticles incorporating poly(d, l-lactide)-block-poly(ethylene glycol)-block-poly(d, l-lactide) to model the mesenchymal stem cells "recruitment-osteogenic" cascade for periodontal bone regeneration [185]. Nanotherapeutic strategies will provide more opportunities for the treatment of periodontitis complications.

### Nanotherapeutic potential of natural active ingredients

Antibiotics and non-steroidal anti-inflammatory agents have potential problems in the treatment of periodontitis including drug resistance, dysbacteriosis and gastrointestinal adverse effects. Natural components derived from natural sources are currently attracting interest from researchers. Natural ingredients with proven therapeutic effects on periodontitis include quercetin, resveratrol, baicalin, curcumin, etc. [187].

Quercetin has a potential protective effect against chronic inflammation-related periodontitis by suppressing the Akt/AMPK/mTOR pathway [188]. In another study, quercetin was found to reduce alveolar bone loss by inhibiting inflammation in periodontitis rats [189]. Resveratrol protects against periodontitis-induced tissue damage by augmenting HO-1 via Nrf2-mediated signaling [190]. Curcumin significantly reduced the expression of TNF-α and IL-6 by inhibiting the phosphorylation of p38 MAPK and reducing the inflammatory response in macrophages [191]. Plumbagin down-regulating the mRNA expression of the pro-inflammatory cytokines TNF-α, IL-1β and IL-6 in periodontium, thereby retarding the development of inflammation [192].

Natural ingredients have achieved excellent experimental results in the treatment of periodontitis. However, most of the natural ingredients have poor solubility and safety problems. In addition, little is known about the in vivo pharmacokinetic studies of natural active ingredients, which limits the clinical translation of natural active ingredients in the treatment of periodontitis. The use of advanced nanodrug delivery systems is expected to solve the problems of drug formation of natural ingredients in the future.

## Conclusion

The in-depth study of pathogenesis and the development of nanomaterial engineering promote the development of nanotherapeutic strategies for periodontitis. The diverse physicochemical properties and targeting properties of nanodelivery systems create a favourable platform for drug delivery to treat periodontitis. In this article, we review the nanotherapeutic strategies for periodontitis to provide inspiration for future advances in periodontitis treatment and innovations in the design of nanodelivery systems. Overall, nanotherapeutics have shown great potential at preclinical levels, but their clinical performance remains to be evaluated. More work is needed to refine the development of novel nanotherapeutic strategies. We believe that nanotherapeutic strategies will soon provide new opportunities for the treatment of periodontitis, thereby alleviating patient suffering and the medical burden on society.

## Data Availability

Not applicable.

## Abbreviations

ABC: Alveolar bone crest
ALP: Alkaline phosphatase
Au NBPs: Au nano bipyramids
AuNPs: Au nanoparticles
BMDM: Bone marrow derived macrophage
BMSCs: Bone marrow stem cells
BV/TV: Bone volume/tissue volume
C5aR: Component 5a receptor
Ce6: Chlorin e6
CEJ: Cement-enamel junction
CeO_2_: Cerium oxide nanoparticles
CXCL: C-X-C motif chemokine ligand
COL-1: Collagen type I
CXCR3: C-X-C motif chemokine receptor 3
DCs: Dendritic cells
Fn: *Fusobacterium nucleatum*
Gel MA: Gelatin methacrylate
ICG: Indocyanine green
IFN‐γ: Interferon‐γ
IL: Interleukin
LPS: Lipopolysaccharide
Micro-CT: Micro computed tomography
NF-κB: Nuclear transcription factor-κB
NIR: Near infrared
OCN: Osteocalcin
OPG: Osteoprotegerin
PDLSCs: Periodontal membrane stem cells
PDT: Photodynamic therapy
Pg: *Porphyromonas gingivalis*
PLGA: Poly (D, L-lactide-co-glycolide)
PTT: Photothermal therapy
RANKL: Receptor activator of nuclear factor kappa-Β ligand
RNA-seq: RNA sequencing
ROS: Reactive oxygen species
RUNX2: Runt-related transcription factor 2
SEM: Scanning electron microscopy
STAT: Signal transducer and activator of transcription
Tb.N: Trabecular number
TEM: Transmission electron microscopy
TGF-β: Transforming growth factor-β
Th-: T helper
TNF-α: Tumor necrosis factor-α
TRAP: Tartrate resistant acid phosphatase
Treg: Regulatory T cell
ZIF-8: Zeolite imidazole skeleton-8
ZnO: Zinc oxide
